# Gestational age and fetal hypothyroidism Alter the porcine thyroid and local hepatic and renal renin‐angiotensin systems

**DOI:** 10.14814/phy2.70565

**Published:** 2025-09-12

**Authors:** Alyssa A. Smith, J. Alex Pasternak

**Affiliations:** ^1^ Department of Animal Sciences Purdue University West Lafayette Indiana USA; ^2^ Department of Animal and Food Sciences University of Kentucky Lexington Kentucky USA

**Keywords:** fetal, hypothyroidism, lectin, local RAS, ontogeny, thyroid

## Abstract

This study was conducted to examine the ontogeny of and interactions between the thyroid system and local renin‐angiotensin systems (RAS) in the porcine fetus. *N* = 24 pregnant gilts were split into two groups, receiving either a daily dose of methimazole to induce fetal hypothyroidism, or a daily sham control. Within each group, treatment of *n* = 3 gilts was initiated at gestational day 34, 45, 55, or 65, and terminated 21 days later to allow for fetal sample collection. The morphology of the fetal thyroid was assessed via lectin staining, showing that follicular structure changes with both increasing gestational age and altered thyroid status. Subsequently, the ontogeny of thyroid and RAS‐related genes in the fetal liver and kidney, as well as the effect of fetal hypothyroidism, was assessed via qPCR. In the liver, *SERPINA7*, *TTR*, *THRA*, and *THRB* were all ontogenically regulated, with *THRB* also ontogenically regulated in the kidney. All studied RAS genes were ontogenically regulated in at least one of the studied tissues. Hypothyroidism caused temporal dysregulations in the expression of hepatic *SERPINA7*, *AGT*, *ACE*, *ACE2*, and *AGTR2*, as well as renal *THRB*, *ACE*, and *AGTR1*. These results suggest a temporal and tissue‐dependent relationship between the thyroid and RAS in the fetal pig.

## 1 INTRODUCTION

Fetal development is contingent on successful activation and function of a variety of endocrine pathways, including the hypothalamic–pituitary–thyroid (HPT) axis and the renin–angiotensin system (RAS). In the human fetus, the HPT axis becomes active early in gestation (Kratzsch & Pulzer, 2008), stimulating the fetal thyroid gland to produce thyroid hormones that are needed for proper in utero development. Similarly, thyroid hormones are first detected in porcine serum around 40% of the way through gestation, after which levels continue to increase as gestation progresses (Brzezińska‐Slebodzińska & Slebodziński, 2004). Notably, the thyroid is the most commonly dysregulated fetal endocrine gland (Ghirri et al., 2016), with deficits in fetal thyroid hormone production resulting in a wide array of detriments, including impaired skeletal development (Hopkins & Thorburn, 1972; Lanham et al., 2011), cognitive deficits (Oerbeck et al., 2003), altered cell cycle regulation (Smith et al., 2024), and dysregulations in other endocrine systems (Letizia et al., 1994; Näntö‐Salonen et al., 1993).

Among these other endocrine systems is the RAS, which is a complex endocrine pathway responsible for regulating both blood volume and blood pressure, primarily through the terminal peptide angiotensin II (ANGII). Canonically, ANGII is systemically produced from hepatic‐derived angiotensinogen (AGT) through a series of enzymatic cleavages involving renal renin (REN) and a more widely expressed angiotensin I converting enzyme (ACE), with ANGII ultimately inducing vasoconstriction via binding to its primary receptor, the angiotensin II receptor type 1 (AGTR1). In addition to AGTR1, ANGII may instead interact with a non‐canonical receptor, AGTR2, to induce countervailing vasodilation (Carey et al., 2001), with an additional vasodilatory action also occurring in response to angiotensin‐(1–7) (ANG‐(1–7)), which is produced following cleavage of ANG peptides by angiotensin converting enzyme 2 (ACE2) (de Moraes et al., 2017). Collectively, the RAS plays a critical role in regulating both maternal and fetal vascular function, and is dynamically regulated throughout the course of pregnancy (Carbone et al., 1993; Forhead et al., 2000; Olson et al., 1990; Robillard et al., 1995; Yang et al., 2013).

In addition to the previously described systemic RAS, tissue‐specific local RAS have also been suggested to operate in a variety of organs. While what exactly constitutes a local RAS is disputed, a general view is that a local RAS occurs when a tissue is able to locally produce ANGII for autocrine or paracrine action (Campbell, 2014). While a local RAS may function via the canonical cleavage of AGT by endogenously produced REN and ACE, some tissues may rely on non‐canonical mechanisms to produce ANGII. These non‐canonical mechanisms include the use of a REN/prorenin receptor termed ATP6AP2 (Nguyen et al., 2002) or cleavage of ANG peptides by alternative enzymes (Izumi & Iwao, 2013). These mechanisms may be especially important for the establishment of a local RAS in tissues that lack the ability to endogenously produce REN or ACE. The existence of local RAS has been demonstrated in a wide array of fetal organs, including the fetal lung (Nogueira‐Silva et al., 2012), ovary (Pountain et al., 2010), heart, brain, and kidneys (Tang et al., 2020). Dysregulations of these local RAS may contribute to disorders affecting fetal development; however, the exact developmental importance of local RAS systems is still unclear, considering the difficulty associated with distinguishing local RAS function from systemic activity (Campbell, 2014). Despite this, studies of RAS in organs such as the liver and kidneys may provide valuable insights into both systemic and local RAS function, considering the canonical role of these tissues in producing AGT and REN, respectively.

Dysregulations in the fetal RAS may occur due to various factors such as maternal undernutrition (Vieira‐Rocha et al., 2022) or consumption of ACE inhibitors, the latter of which is a known teratogen for organs such as the kidneys (Cooper et al., 2006; Plazanet et al., 2014; Tabacova et al., 2003). While the mechanism by which disruptions in the RAS lead to teratogenicity is not entirely understood, this suggests a potential role of the RAS in fetal development, which is of particular interest considering that hypothyroidism is known to dysregulate the RAS (Marchant et al., 1993; Vargas et al., 2012) and also impair fetal development through relatively unknown mechanisms. Additionally, multiple components of the fetal RAS (Carbone et al., 1993; Olson et al., 1990) and fetal thyroid (Allen et al., 1995; Brzezińska‐Slebodzińska & Slebodziński, 2004; Devaskar et al., 1986) are known to exhibit concomitant increases in activity throughout gestation, suggesting that thyroid hormones may partially regulate the RAS. The endocrine interaction between thyroid hormones and the RAS has been partially described in a small number of studies involving ovine (Chen et al., 2005) and murine (Matilla et al., 1993) models of fetal hypothyroidism, and has also been observed postnatally in human cases of congenital hypothyroidism (Letizia et al., 1994). However, to the author's best knowledge, the relationship between thyroid hormones and the RAS throughout gestation is yet to be directly examined in the porcine fetus, which is becoming increasingly recognized as a critical biomedical model for human development (Lunney et al., 2021). Similarly, little is known about the local production of, or the ontogenic regulation of, RAS components in porcine fetal tissues throughout gestation.

To address these knowledge gaps, the objectives of the present study were two‐fold: first, to examine the ontogenic trajectory of the porcine thyroid system and local hepatic and renal RAS; and second, to determine the temporal interactions between these systems. We hypothesized that the fetal thyroid (ROID) and the expression of genes regulating thyroid hormone bioactivity would change both throughout gestation and in response to fetal thyroid status. Further, we hypothesized that local production and expression of RAS components would be observed in the fetal liver (LVR) and kidney (KID), with gene expression differentially regulated throughout gestation, and hypothyroidism resulting in dysregulations of these genes. To examine these hypotheses, we utilized a temporal model of porcine fetal hypothyroidism to first assess morphological changes occurring in the fetal ROID, and then to assess the transcriptional ontogeny and response to fetal hypothyroidism of key genes regulating thyroid hormone bioactivity and RAS function in the fetal LVR and KID.

## 2 MATERIALS AND METHODS

### 2.1 Animal model and sample collection

For immunohistofluorescence (IHF), samples of fetal liver (LVR) and kidney (KID) were collected from *n* = 4 euthyroid porcine fetuses at gestational day (GD) 96, all of which were terminal crosses derived from a single commercial crossbred gilt (Yorkshire × Landrace) that was euthanized by penetrating captive bolt followed by exsanguination. These samples were fixed in 10% neutral buffered formalin and paraffin‐embedded for histology by the Purdue Histology Research Laboratory.

All other samples utilized in this study were derived from an animal experiment that has previously been described in detail (Smith & Pasternak, 2025). This experiment involved 24 commercial crossbred gilts (Yorkshire × Landrace), which were selected from the Purdue University Animal Sciences Research and Education Center. Following selection, gilt estrous cycles were synchronized with a 14‐day course of 17.6 mg Altrenogest administered daily in feed (Merck Animal Health, Kenilworth, NJ, USA), and gilts bred with pooled Duroc semen via artificial insemination in the first standing estrus following withdrawal. Gilts were confirmed pregnant by transcutaneous ultrasound around GD 30, and subsequently subdivided into four groups, with *n* = 6 gilts beginning a 21‐day course of daily treatment at GD 34, 45, 55, or 65 ± 1 of the normal 114‐day gestational period. These timepoints were chosen such that the overall study period spanned from prior to the onset of fetal thyroid function to the beginning of late gestation, with the 21‐day treatment period originally established as a non‐pathogenic model for mimicking thyroid hormone suppression occurring in virally infected swine fetuses (Pasternak et al., 2020b). Within each timepoint, *n* = 3 gilts were randomly assigned to receive either a daily oral dose of 5 mg/kg methimazole (MMI) (Cat. No. M8506; Sigma‐Aldrich, St. Louis, MO, USA) dissolved in 50% corn syrup/50% water and administered in supplemental feed to induce fetal hypothyroidism, or an equivalent sham control (CON), as previously described in late gestation swine (Ison et al., 2023). Throughout the treatment period, the gilts were weighed twice weekly to allow for an appropriate adjustment of treatment dosages.

Following 21 days of treatment, the gilts from each group were humanely euthanized by penetrating captive bolt and exsanguination at GDs 55, 66, 76, and 86 ± 1, respectively. Of importance to the studied tissues, these timepoints are characterized by very high tissue levels of thyroxine in the LVR and KID (Krysin, 1995), which may suggest an increased importance of thyroid hormones for the development of these tissues during this period of gestation. Following euthanasia of the dam, a midline ventral incision was made to allow for the removal of the gravid uterus, and tissue samples were collected from the two most centrally located, viable male and female fetuses from each uterine horn (*n* = 4 fetuses/gilt), for a total of *n* = 96 fetuses in the overall sample population. Tissue samples, including a distal portion of fetal LVR, a portion of fetal KID, and the fetal thyroid (ROID) were collected, with the LVR and KID flash frozen in liquid nitrogen and stored at −80°C, and the ROID fixed in 10% neutral buffered formalin and paraffin embedded for histology by the Purdue Histology Research Laboratory. All animal procedures were carried out in compliance with Purdue University's animal care policies and approved by the Institutional Animal Care and Use Committee (IACUC Protocol #0123002344).

### 2.2 Thyroid lectin staining

To allow for a quantitative assessment of ROID ontogeny, as well as an assessment of the temporal severity of induced hypothyroidism, the formalin‐fixed paraffin embedded fetal ROIDs were lectin‐stained for histological assessment. Initially, a panel consisting of seven fluorescein‐conjugated lectins (Cat. No. FLK‐2100; Vector Laboratories, Newark, CA, USA) was evaluated using a method previously described in other tissues (Gewaily et al., 2021). In short, histology sections were independently stained with either 5 μg/mL wheat germ Agglutinin (WGA), 10 μg/mL concanavalin A (Con A), or 20 μg/mL dolichos biflorus agglutinin (DBA), peanut agglutinin (PNA), ricinus communis agglutinin I (RCA120), soybean agglutinin (SBA), or ulex europaeus agglutinin I (UEA1). Following the panel test, RCA120 was selected to stain a larger subset of samples consisting of *n* = 48 fetal ROIDs (3 males and 3 females from each treatment group at each GD), which were sectioned at 5 μm on a HM 325 rotary microtome and mounted on slides. Prior to staining, slides were dried at 60°C for 15 min, deparaffinized, and rehydrated through a decreasing gradation of ethanol. Subsequently, sections were blocked for 1 h at room temperature in 1% bovine serum albumin (BSA) diluted in phosphate‐buffered saline (PBS) and then incubated in 20 μg/mL RCA120 for 1 h at room temperature. Counterstaining was accomplished by incubation in 0.1 μg/mL 4',6‐diamidino‐2‐phenylindole (DAPI) (Thermo Fisher, Waltham, MA, USA) for 10 min, and sections cover slipped with Mowiol 4–88 mounting medium (Sigma‐Aldrich).

After drying of the mounting medium, sections were imaged on an ECHO Revolution microscope (ECHO, San Diego, CA, USA) using a 20× objective. For the initial panel, one representative image was taken from each section, with exposure times kept consistent across all images. For the larger subset stained with RCA120, 2–6 images were taken per sample based on the number of unique artifact‐free areas that could be identified (average of 5.54 images per sample). Exposure for the channel representing RCA120 signal was kept consistent for all samples, and resultant images were assessed using an automated macro in ImageJ (Schneider et al., 2012). Briefly, images were binarized for particle analysis, with requirements for threshold, follicle circularity, and follicle area determined in preliminary experiments and universally applied to all images. Output values generated for each individual image included the percentage of image area comprised of follicles, follicle count, mean follicle circularity, mean follicle area, and mean staining intensity, with replicate images for each sample averaged to give a final value for later analysis.

### 2.3 Relative gene expression assessment

To allow for assessment of gene expression, flash‐frozen tissue samples of the fetal LVR and KID were processed for RNA extraction, reverse transcription, and RT‐qPCR. Samples were first manually ground to a fine powder under liquid nitrogen, and RNA was extracted using TRIzol reagent (Cat. No. 15596026; Thermo Fisher). DNA contamination was removed using the Turbo DNA‐free kit (Cat. No. AM1907; Thermo Fisher) with the addition of 20 IU RNaseOUT (Cat. No. 10777019; Thermo Fisher) in each reaction. RNA purity and concentration, as well as integrity, were assessed via a Nanodrop ND1000 (Thermo Fisher) and denaturing agarose gel, respectively. For each sample, 2 μg of total RNA was reverse transcribed using the High‐Capacity cDNA Reverse Transcription Kit (Cat. No. 4368813; Applied Biosystems, Foster City, CA, USA) with the addition of 20 IU RNaseOut to prevent sample degradation. Reaction products were diluted with nuclease‐free water to 10 ng/μL and stored at −20°C. Due to sample loss of one LVR sample from the CON group at GD 55 and one from the CON group at GD 76, the final sample number for these groups was *n* = 11, with *n* = 12 for all other groups.

Primers for RT‐qPCR reference genes and *AGT* were derived from prior literature, while primers for all other genes of interest (GOIs) (Table 1) were designed based on the Sscrofa 11.1 genome assembly to span exon‐exon junctions (where possible) as identified using the BLAST‐like alignment tool (BLAT). Specificity was confirmed using the Basic Local Alignment Search Tool (BLAST), and primer sequences were confirmed to target all known and predicted transcript variants in the pig. Primers were experimentally validated to confirm the production of a single amplicon product, with all primers additionally determined to have an efficiency between 90% and 110% as assessed by a 5‐point serial dilution. For RT‐qPCR, validated primers were used at 0.33 μM in reaction with 20 ng cDNA and SSoAdvanced Universal SYBR Green Supermix (Cat. No. 1725274; Bio‐Rad, Hercules, CA, USA), with reactions ran in duplicate on a CFX Connect qPCR system (Bio‐Rad), and the coefficient of variance between all duplicate samples was equal to or less than 1%.

**TABLE 1:** Porcine specific primer sequences used for RT‐qPCR.

	Gene ID	Symbol	Forward primer	Reverse primer	Amplicon length (bp)	Annealing temp (°C)	Target sequence/reference
Reference Genes	414396	*ACTB*	5′‐CCAGCACGATGAAGATCAAG‐3′	5′‐AGTCCGCCTAGAAGCATTTG‐3′	171	61	Ison et al. (2023)
	396989	*RPL19*	5′‐AACTCCCGTCAGCAGATCC‐3′	5′‐AGTACCCTTCCGCTTACCG‐3′	147	61	Pasternak et al., (2015)
	780440	*YWHAZ*	5′‐TGATGATAAGAAAGGGATTGTGG‐3′	5′‐GTTCAGCAATGGCTTCATCA‐3′	203	60	Pasternak et al., (2020b)
	100628048	*STX5*	5′‐TGCAGAGTCGTCAGAATGGA‐3′	5′‐CCAGGATTGTCAGCTTCTCC‐3′	144	61	Pasternak et al., (2020a)
Genes of Interest	397125	*SERPINA7*	5′‐GAAATGGAACCGCTTACTGC‐3′	5′‐GGGCAGCATTGGAAAGTTTA‐3′	182	60	NM_214058.2
	397419	*TTR*	5′‐TGAACGTAGGCGTGAAAGTG‐3′	5′‐TGGCTGTGAACACAACCTCT‐3′	216	60	NM_214212.1
	397387	*THRA*	5′‐ACAGTGCCAGGTCACCAGAT‐3′	5′‐CTGCTCGTCTTTGTCCAGGT‐3′	107	60	NM_214190.1
	396776	*THRB*	5′‐CCAATAGTAAACGCCCCAGA‐3′	5′‐CACATGGCAGCTCACAAAAC‐3′	136	62	XM_013981418.2
	100157073	*AGT*	5′‐GCACTTCCAAGGAAAGGTGA‐3′	5′‐CGACACTGAGGTGGTGTTGT‐3′	82	60	Pasternak et al., (2020b)
	100524822	*REN*	5′‐ATCCTTTCCCAGAAGGTGCT‐3′	5′‐CCTCACAGACACCCCTTTCA‐3′	186	60	XM_021063212.1
	100514935	*ATP6AP2*	5′‐CTGGCAGGTTTGGATGAAAT‐3′	5′‐CTTGGTGGGTCCTTCACTTG‐3′	227	60	XM_003135022.4
	613133	*ACE*	5′‐GGAGCCTGATCTGACAAACC‐3′	5′‐TCCATCTTCGTAGCCATTGA‐3′	166	61	NM_001033015.3
	100144303	*ACE2*	5′‐ACAGGGACAGGGGACTATGA‐3′	5′‐CACATATCGCCAAGCAAATG‐3′	185	60	NM_001123070.1
	100519976	*AGTR1*	5′‐ATAAATGCCATCCTCCCAGTC‐3′	5′‐TACTGCCCTTTGGAAATTGG‐3′	140	60	XM_003132469.4
	733586	*AGTR2*	5′‐TTCTCCTGGGATTCACCAAC‐3′	5′‐TCGGCATGACACACTCTCTC‐3′	140	62	XM_005673847.3

### 2.4 Immunohistofluorescence

To examine the spatial localization of locally produced RAS components in the fetal LVR and KID, IHF was performed using rabbit polyclonal IgG antibodies for AGT (Cat. No. PA5‐21520), ACE (Cat. No. PA5‐86568), and ACE2 (Cat. No. PA5‐85139) (Invitrogen, Thermo Fisher). Antibodies were first determined to have specificity for each target protein as assessed by Western Blots containing porcine fetal LVR and KID protein lysates (Figure S1). In the fetal LVR and KID, the detected band for AGT was well below the expected molecular weight of the full‐length protein; however, this band was also observed in the HepG2 positive control (Figure S1a) and has a molecular weight consistent with a non‐ANG fragment resulting from REN cleavage at additional leucine‐valine pairs. Bands for ACE were present at the expected molecular weight in both tissues (Figure S1b). The band for full‐length ACE2 was only detected in the KID, with the smaller molecular weight band observed around 58 kDa in both tissues indicative of a truncated form of ACE2 that has previously been identified in the pig (Figure S1c) (Zhang et al., 2021). Following Western Blots, antibodies were validated for IHF to determine the optimal concentration for staining using a series of concentrations relative to a no primary and isotype control. Once validated, staining was conducted on 5 μm sections of formalin‐fixed paraffin‐embedded fetal LVR and KID samples that were cut on a HM 325 rotary microtome, with *n* = 3 unique samples stained for each target in each tissue.

Tissue sections were dried, deparaffinized, and rehydrated as described above. Subsequently, heat‐mediated antigen retrieval was performed for 30 min at 95°C in sodium citrate buffer pH 6.0 containing 0.05% Tween 20. Slides were then washed in PBS and blocked for 2 h at room temperature in an incubation buffer consisting of 1% horse serum, 0.5% Triton X‐100, 0.02% sodium azide, and 2% BSA diluted in PBS. Slides were incubated overnight at 4°C in primary antibody diluted to working concentrations of 10 μg/mL for AGT, 20 μg/mL for ACE, and 2 μg/mL for ACE2. On day two, slides were washed before incubating with 2 μg/mL of a goat anti‐rabbit IgG Alexa Fluor 647‐conjugated secondary antibody (Cat. No. 4010‐31; SouthernBiotech, Birmingham, AL, USA) for 2 h at room temperature. Slides were then counterstained with DAPI, washed with PBS, and cover slipped with Mowiol 4–88 mounting medium. All imaging was conducted on an ECHO Revolution microscope, with entire tissue sections tile scanned with a 10× objective. Resultant grayscale images were processed in ImageJ. Images were first background subtracted, and the display range adjusted consistently within each tissue and target to allow for easy visualization. Subsequently, the images were cropped to a representative area, pseudocolored, and merged for presentation.

### 2.5 Statistical analyses

All statistical analyses were performed in R version 4.3.1 (R Core Team, 2023), with plots generated using the ggplot2 package (Wickham, 2016). For assessment of the fetal ROIDs using RCA120 staining, data was fit to a linear model including GD, treatment group, and the interaction. Normality of the residuals was visually assessed with a quantile‐quantile plot, with the data for mean follicle area log transformed prior to analysis to improve normality. One MMI fetus at GD 76 had no follicles detected across any of the images taken and was removed from the analyses for mean follicle area, mean staining intensity, and mean follicle circularity. Pairwise contrasts were performed using the emmeans package (Lenth, 2024) with a Šidák correction to first assess the ontogenic changes occurring across time within the CON group, and then to determine the impact of hypothyroidism within each individual timepoint.

Consistent with prior literature, the stability of canonical reference genes could not be demonstrated with increasing gestational age. As such, RT‐qPCR data was analyzed parametrically with the residual correction method as previously described (Novak et al., 2012; Smith & Pasternak, 2025; Vallet et al., 2010). Following assessment of various reference genes, the geometric means of the three most stable reference genes within each tissue (*ACTB*, *YWHAZ*, and *RPL19* in the LVR; *ACTB*, *YWHAZ*, and *STX5* in the KID) were fit to a linear model including GD, treatment group, and the interaction, and the residuals extracted and used as correction factors to calculate ΔCt values for each GOI. The ΔCt values for each gene in each tissue were then fit to a linear model including GD, treatment group, and the interaction, and the model emmeans and 95% confidence intervals converted to fold changes using the 2^−ΔΔCt^ method to allow for graphing. Model pairwise contrasts were performed using the emmeans package (Lenth, 2024) with a Šidák correction to first establish the ontogenic trajectory of gene expression within the CON group, and then to determine the temporal impact of hypothyroidism on expression within each timepoint.

For all presentation of data extracted from linear models, the CON groups are connected with solid lines to allow for easy visualization of ontogenic changes, while the MMI groups are instead represented with dotted lines as no direct ontogenic assessment was made across time within the MMI group. For all analyses, the threshold for statistical significance was *p* < 0.05, with results between 0.05 < *p* < 0.10 reported as trends.

## 3 RESULTS

### 3.1 Thyroid histology

Representative images of thyroid staining for the seven initially tested lectins are shown in Figure S2a,b. Visual assessment of staining patterns in CON fetuses at GDs 55 and 86 showed that Con A, RCA120, and WGA all stained ROID follicles, with no marked signal detected for staining with DBA, PNA, SBA, or UEA1. The signal for WGA appeared stronger around the periphery of the follicle, consistent with the apical brush border of the thyrocytes. In contrast, both Con A and RCA120 uniformly stained the whole colloid. However, RCA120 was more specific to the colloid and had minimal background signal, so it was thus chosen for staining the larger subset of fetuses to generate quantitative morphological data.

**FIGURE 1:**
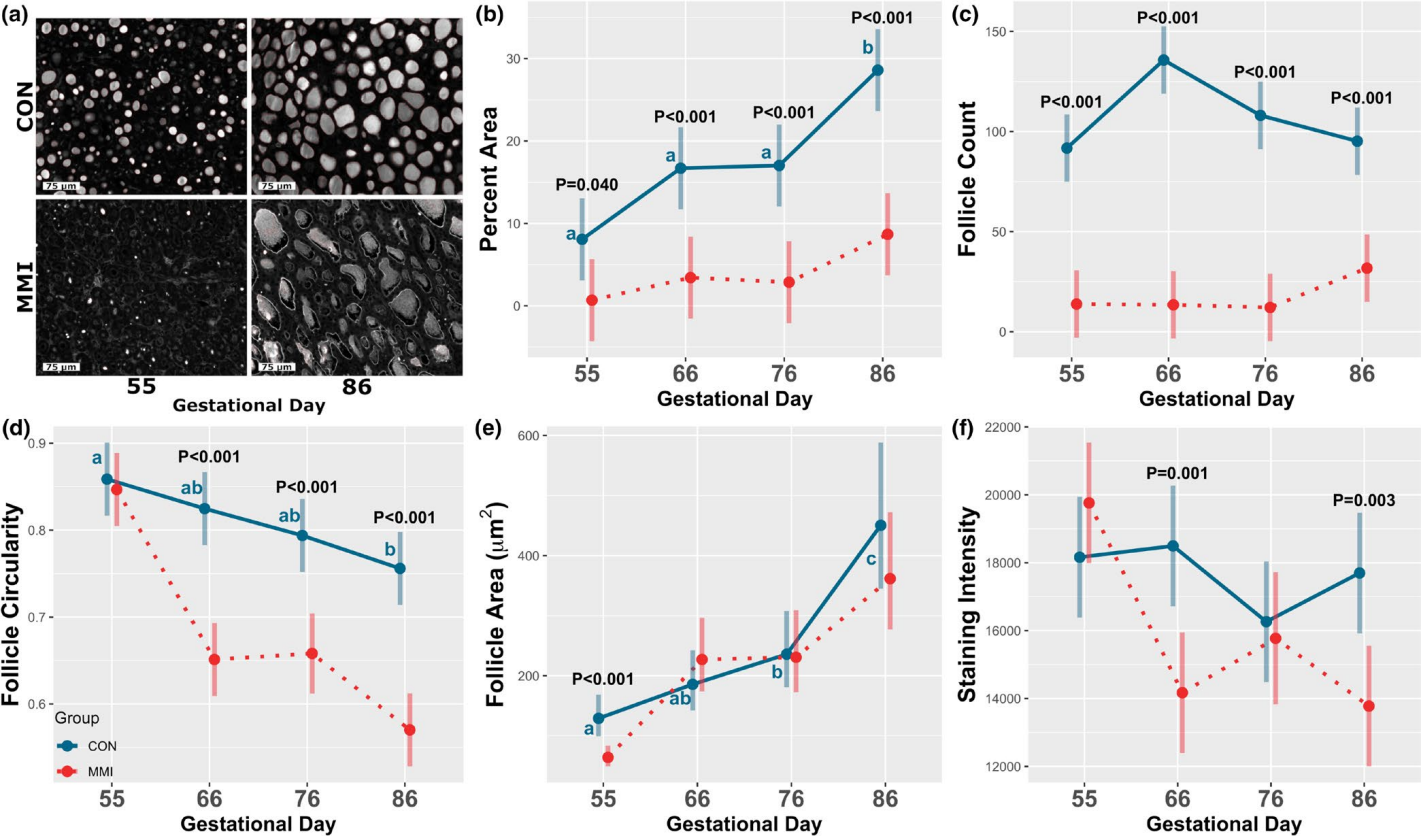
Fetal thyroid ontogeny. (a) Example images showing staining of CON and MMI fetal thyroids at gestational days 55 and 86, with detected follicular regions outlined in red. (b) Percentage of image area comprised of follicles, (c) mean follicle count, (d) mean follicle circularity, (e) mean follicle area, (f) and mean follicle staining intensity of detected regions in fetal thyroids (*n* = 6/group) following 21 days of maternal CON or MMI treatment at various timepoints throughout gestation. Unique letter superscripts denote statistically significant (*p* < 0.05) changes in the CON group over time, with statistically significant differences between the CON and MMI group within each timepoint displayed numerically. Data are presented as the estimated marginal means with 95% confidence intervals, as extracted from a linear model including gestational day, treatment group, and the interaction.

While in CON fetuses, RCA120 intensely and consistently stained the follicular colloid; staining was largely absent in the MMI ROIDs at GD 55 and appeared irregular in the MMI ROIDs at GD 86 (Figure 1a). Among the CON groups, the mean percentage of each image area detected as follicle (percent area), mean follicle circularity, and mean follicle area were all significantly altered with increasing gestational age (Figure 1b,d,e). Specifically, both percent area and follicle area were significantly increased at GD 86 relative to all other GDs (*p* < 0.001, *p* = 0.009, and *p* = 0.011 for percent area at GDs 55, 66, and 76, respectively; *p* < 0.001, *p* < 0.001, and *p* = 0.008 for follicle area at GDs 55, 66, and 76, respectively). Follicle area was additionally increased at GD 76 relative to GD 55 (*p* = 0.015), with a trend observed towards increased percent area between GDs 55 and 76 (*p* = 0.081). In contrast, follicle circularity was significantly decreased at GD 86 relative to GD 55 only (*p* = 0.007). The other measured parameters, mean follicle count and the mean staining intensity of detected regions, were not significantly altered in response to increasing gestational age (Figure 1c,f).

Both percent area and follicle count were significantly decreased in the MMI fetuses relative to age‐matched CONs at all four timepoints (*p* = 0.040, *p* < 0.001, *p* < 0.001, and *p* < 0.001 for percent area at GDs 55, 66, 76, and 86, respectively; *p* < 0.001 for all comparisons for follicle count), while follicle circularity was decreased in the MMI fetuses at GDs 66, 76, and 86 only (*p* < 0.001 for all comparisons). Staining intensity was significantly decreased in the MMI fetuses relative to CON at GDs 66 and 86 only (*p* = 0.001 and *p* = 0.003, respectively), while, in contrast, follicle area was only decreased in the MMI fetuses at GD 55 (*p* < 0.001). There was a significant GD by treatment group interaction observed for follicle count, follicle circularity, follicle area, and staining intensity (*p* = 0.007, *p* < 0.001, *p* = 0.011, and *p* = 0.004, respectively), with no significant interaction effect observed for percent area.

### 3.2 Expression of genes regulating thyroid hormone bioactivity

**FIGURE 2:**
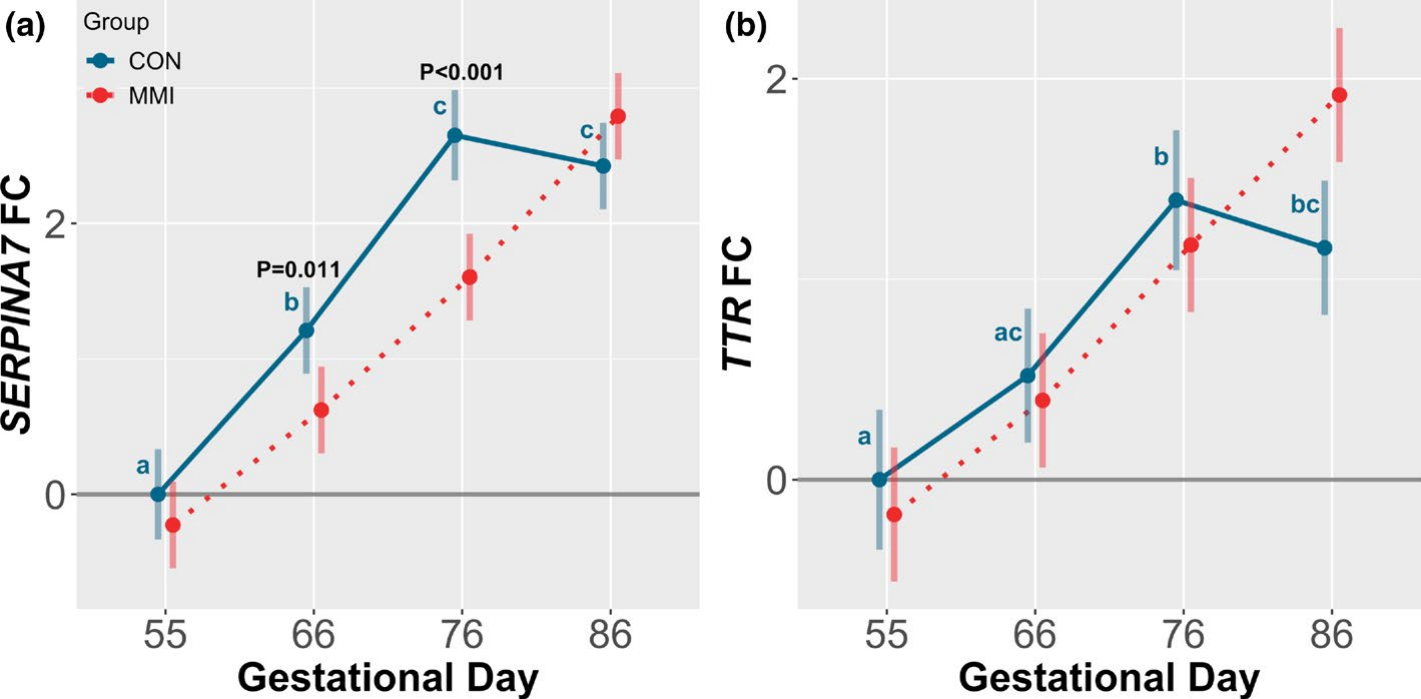
Gene expression of thyroid hormone binding proteins in fetal liver (LVR). Expression of (a) *SERPINA7* and (b) *TTR* in fetal LVR tissue derived from fetuses (*n* = 11–12/group) at various gestational timepoints following 21 days of maternal CON or MMI treatment. All fold changes (FC) were calculated relative to the CON group at gestational day 55, with unique letter superscripts denoting statistically significant (*p* < 0.05) changes in gene expression in the CON group over time, and statistically significant differences between the CON and MMI groups within each timepoint displayed numerically. Data are presented as the estimated marginal means with 95% confidence intervals, as extracted from a linear model including gestational day, treatment group, and the interaction.

In the LVR, both assessed genes encoding thyroid hormone binding proteins, serpin family A member 7 (*SERPINA7*) and transthyretin (*TTR*) (Figure 2a,b), followed a similar trajectory of increasing expression throughout gestation within the CON group. *SERPINA7* was significantly upregulated at GDs 66, 76, and 86 relative to GD 55 (*p* < 0.001 for all comparisons), with expression also significantly higher at GDs 76 and 86 relative to GD 66 (*p* < 0.001 for both comparisons). Similarly, *TTR* was significantly upregulated at GDs 76 and 86 relative to GD 55 (*p* < 0.001 for both comparisons), and also at GD 76 relative to GD 66 (*p* = 0.003), with an additional trend observed towards upregulation at GD 86 relative to GD 66 (*p* = 0.052). While the expression of *TTR* was not impacted by MMI‐induced fetal hypothyroidism, there was a significant downregulation in the expression of *SERPINA7* in the MMI fetuses at GDs 66 and 76 relative to the equivalent age‐matched CONs (*p* = 0.011 and *p* < 0.001, respectively). Additionally, there was a significant GD by treatment group interaction observed for both *SERPINA7* and *TTR* (*p* < 0.001 and *p* = 0.012, respectively).

**FIGURE 3:**
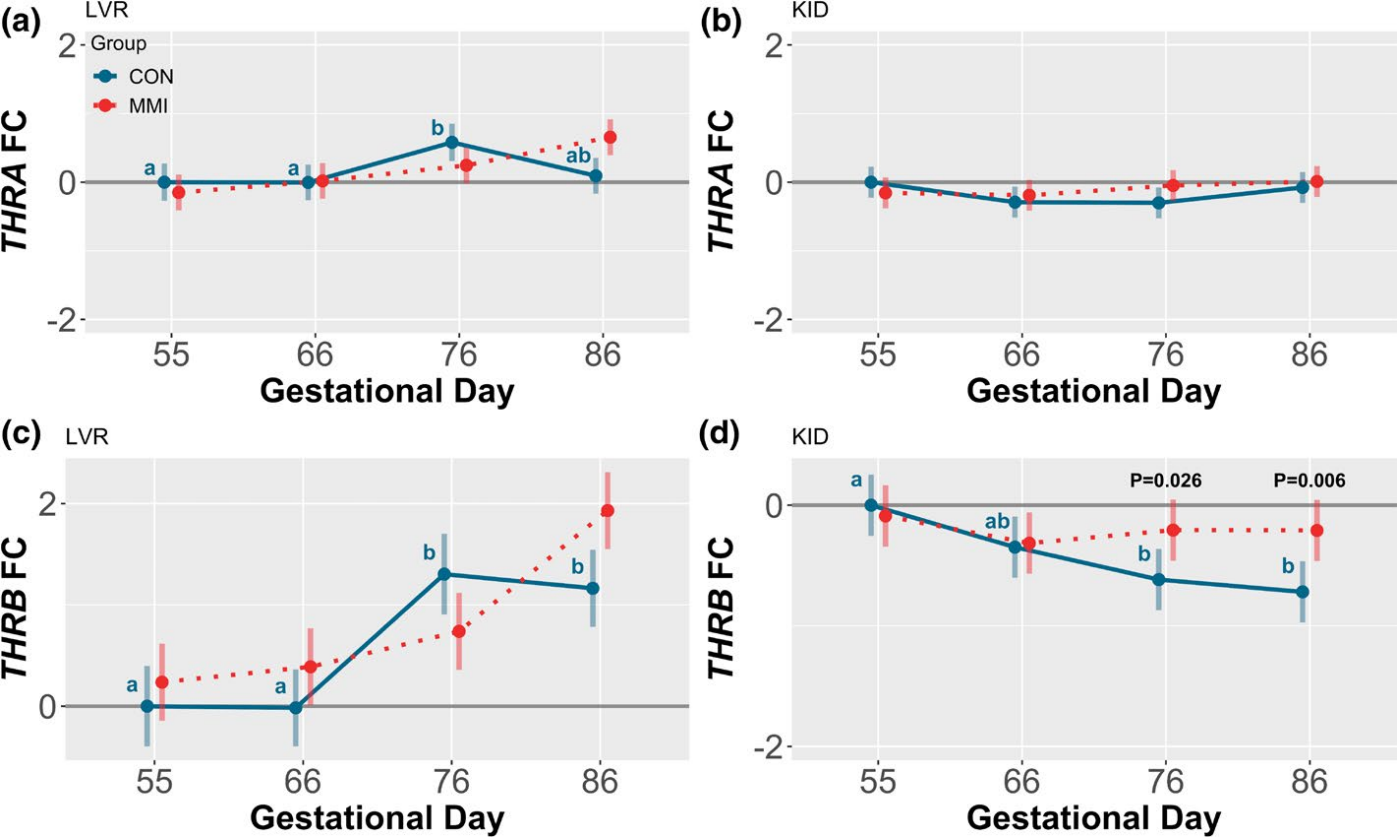
Gene expression of thyroid hormone receptors in fetal liver (LVR) and kidney (KID). Expression of (a, b) *THRA* and (c, d) *THRB* in fetal (a, c) LVR and (b, d) KID tissue derived from fetuses (*n* = 11–12/group) at various gestational timepoints following maternal CON or MMI treatment for a 21‐day period of gestation. All fold changes (FC) were calculated relative to the CON group at gestational day 55, with unique letter superscripts denoting statistically significant (*p* < 0.05) changes in gene expression in the CON group over time, and statistically significant differences between the CON and MMI group within each timepoint indicated by *p*‐values. Data are presented as the estimated marginal means with 95% confidence intervals, as extracted from a linear model including gestational day, treatment group, and the interaction.

Ontogenic alterations were observed in the expression of both hepatic thyroid hormone receptor alpha (*THRA*) and hepatic thyroid hormone receptor beta (*THRB*) among the CON groups (Figure 3a,c), as well as renal *THRB* (Figure 3d), with no significant impact of differing gestational age on renal *THRA* expression (Figure 3b). In the LVR, *THRA* was modestly increased at GD 76 relative to both GDs 55 and 66 (*p* = 0.021 and *p* = 0.016, respectively), while hepatic expression of *THRB* was significantly upregulated at GDs 76 and 86 relative to GDs 55 and 66 (*p* < 0.001 for all comparisons). An additional trend towards downregulation of hepatic *THRA* was also observed at GD 86 relative to GD 76 (*p* = 0.068). In contrast, expression of *THRB* in the KID was decreased at GDs 76 and 86 relative to GD 55 only (*p* = 0.006 and *p* < 0.001, respectively). Within each timepoint, there were minimal effects of MMI treatment on thyroid hormone receptor expression, with only a significant upregulation in renal *THRB* expression observed in the MMI fetuses at GDs 76 and 86 relative to age‐matched CONs (*p* = 0.026 and *p* = 0.006, respectively). Finally, there was a significant GD by treatment group interaction for hepatic expression of both *THRA* and *THRB* (*p* = 0.007 and *p* = 0.008, respectively), with no significant interaction for renal *THRA* expression, and a trend towards interaction significance for renal *THRB* expression (*p* = 0.060).

### 3.3 Hepatic localization of RAS components

**FIGURE 4:**
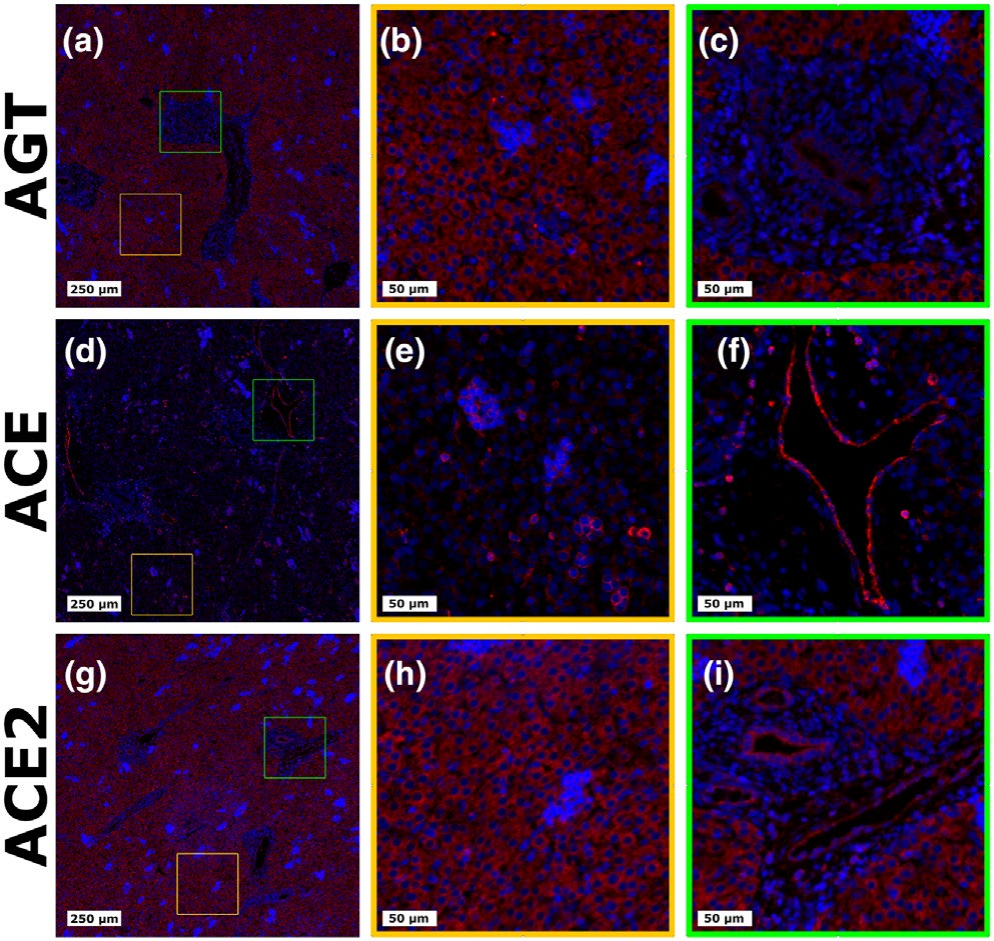
Immunohistofluorescent staining of RAS components in fetal liver. IHF images showing cellular localization of (a–c) AGT, (d–f) ACE, and (g–i) ACE2 in fetal liver tissue derived from porcine fetuses at day 96 of gestation. Target proteins are shown in red and DAPI is shown as a counterstain and colored blue. The leftmost images show large tissue overviews, with colored inset boxes representing the zoomed in sections shown in columns two and three.

AGT, ACE, and ACE2 were all detected in the fetal LVR when assessed by IHF, with representative images shown in Figure 4, and additional examples shown in Figures S3–S5. As expected, staining for AGT (Figure 4a) was cytoplasmic in nature and displayed no apparent spatial specificity, with relatively uniform staining present throughout the cytoplasm of all hepatocytes (Figure 4b), and no marked staining of any structures in the portal triad (Figure 4c). Similarly, the most intense staining for ACE2 (Figure 4g) was observed in the cytoplasm of hepatocytes (Figure 4h), with additional staining noted in cholangiocytes and endothelial cells lining structures in the portal triad (Figure 4i). In contrast to AGT and ACE2, ACE staining (Figure 4d) was localized largely around cellular clusters found stochastically distributed throughout the fetal LVR (Figure 4e), with intense staining of endothelial cells also observed (Figure 4f).

### 3.4 Renal localization of RAS components

**FIGURE 5:**
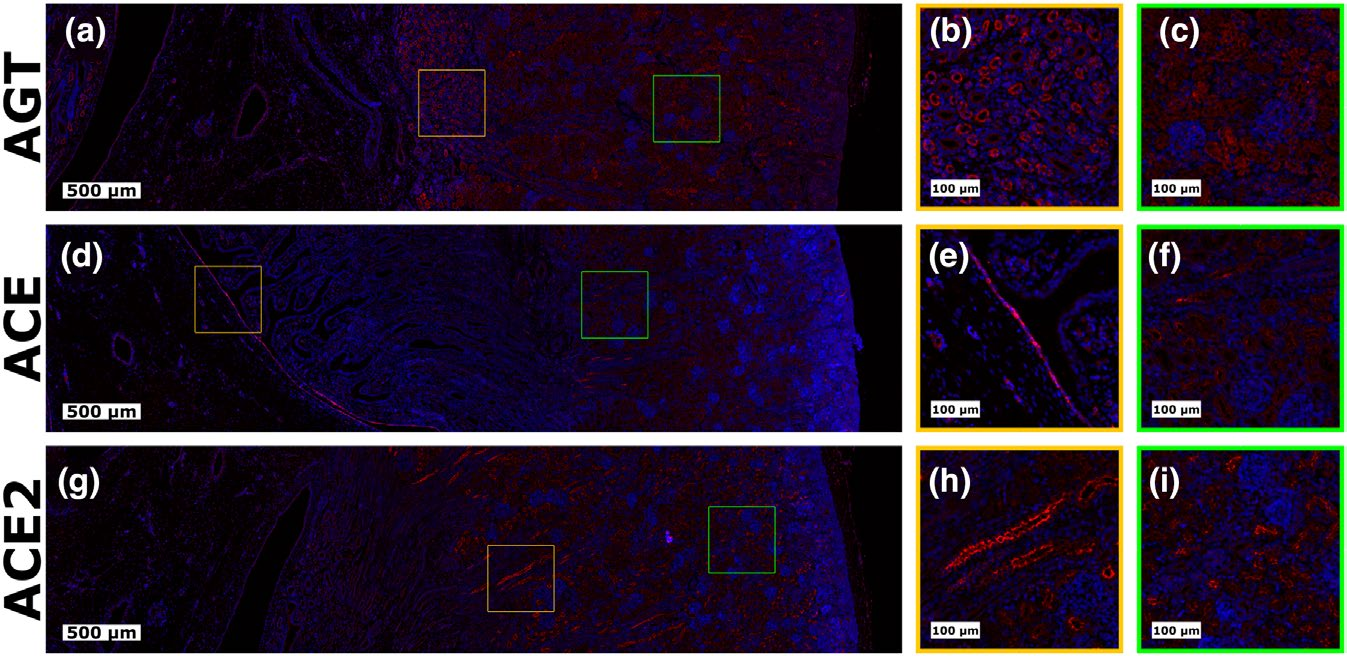
Immunohistofluorescent staining of RAS components in fetal kidney. IHF images showing cellular localization of (a–c) AGT, (d–f) ACE, and (g–i) ACE2 in fetal kidney tissue derived from porcine fetuses at day 96 of gestation, with target proteins shown in red and DAPI shown as a counterstain and colored blue. Leftmost images show large tissue overviews, with colored inset boxes representing the zoomed in sections shown in columns two and three.

In contrast to in the LVR, marked spatial specificity was observed in the localization of all three protein targets in the fetal KID, with representative images shown in Figure 5, and additional examples shown in Figures S6–S8. AGT was found throughout both the cortex and medulla (Figure 5a). In the medulla, AGT was localized to the cytoplasm of epithelial cells lining loops of Henle (Figure 5b). In the cortex, AGT stained the cytoplasm of cells in both the pars convoluta and pars radiata tubules (Figure 5c). In contrast to AGT, both ACE (Figure 5d) and ACE2 (Figure 5g) were localized largely to the cortex region of the KID and absent in the medulla. In the cortex, the most intense staining for ACE was observed on the apical membranes of tubules in the pars radiata, with comparatively weaker apical membrane staining also observed throughout tubules in the pars convoluta (Figure 5f). Additionally, intense ACE staining of the epithelial cells lining the renal pelvis was also observed (Figure 5e). Similar to ACE, staining for ACE2 was most intense on the apical membranes of tubules present throughout the cortex (Figure 5h,i), with no staining observed in the medulla.

### 3.5 Expression of *AGT* and genes contributing to its primary cleavage

**FIGURE 6:**
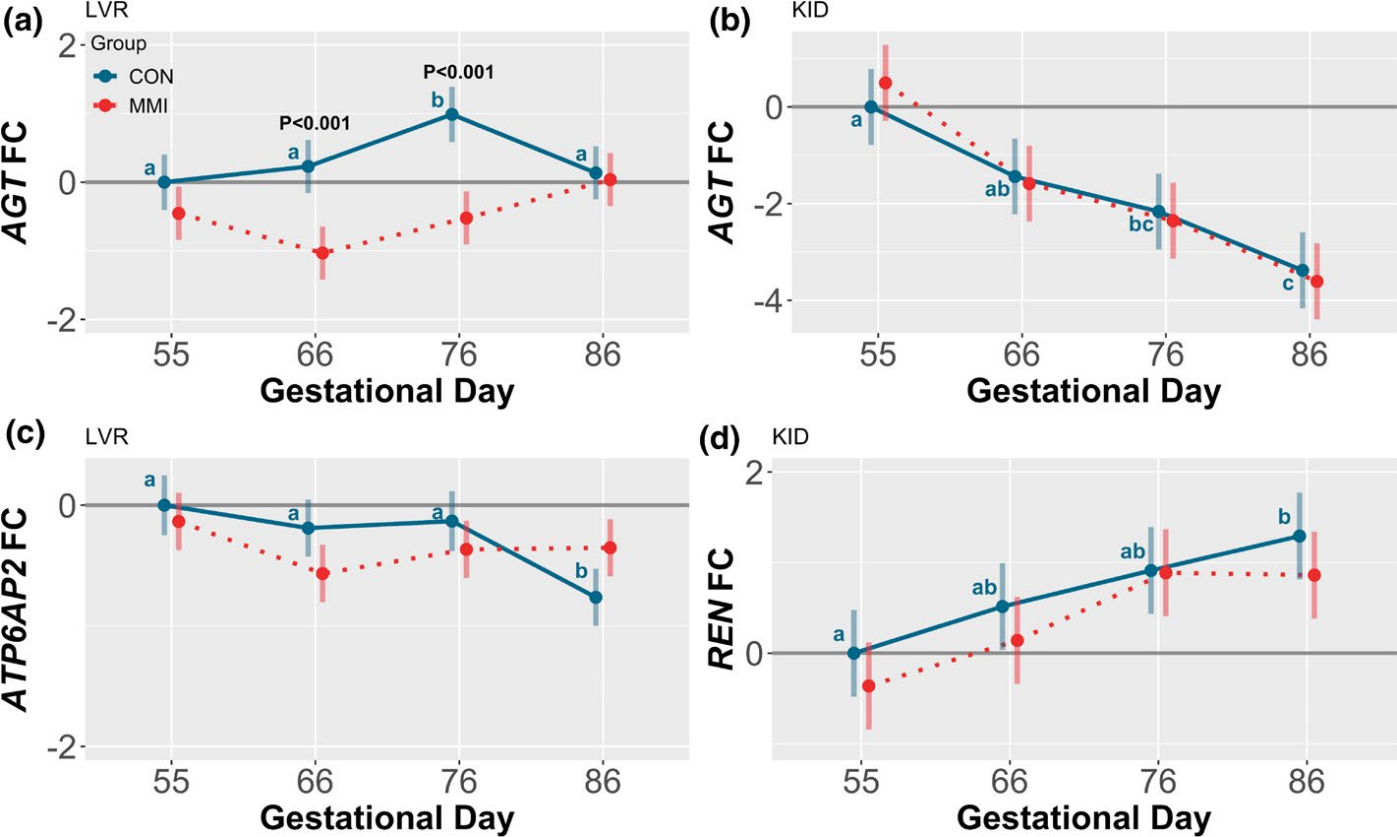
Gene expression of AGT and genes contributing to its primary cleavage in fetal liver (LVR) and kidney (KID). Pregnant gilts were treated for 21 days with either MMI or an equivalent CON administered at various timepoints throughout gestation, and expression of *AGT* in (a) LVR and (b) KID, LVR expression of (c) *ATP6AP2*, and KID expression of (d) *REN* assessed in tissue derived from fetuses (*n* = 11–12/group) at the end of each treatment period. All fold changes (FC) were calculated relative to the CON group at gestational day 55. Unique letter superscripts denote statistically significant (*p* < 0.05) changes in gene expression in the CON group over time, with statistically significant differences between the CON and MMI groups within each timepoint displayed numerically. Data are presented as the estimated marginal means with 95% confidence intervals, as extracted from a linear model including gestational day, treatment group, and the interaction.

Among CON fetuses, expression of hepatic and renal *AGT* (Figure 6a,b) was differentially impacted by the effects of increasing gestational age. Specifically, hepatic expression was significantly upregulated at GD 76 relative to all other timepoints (*p* = 0.005, *p* = 0.0497, and *p* = 0.019 for GDs 55, 66, and 86, respectively), while, in contrast, renal expression was significantly decreased at GDs 76 and 86 relative to GD 55 (*p* = 0.001 and *p* < 0.001, respectively), and also at GD 86 relative to GD 66 (*p* = 0.005). Additionally, a trend was observed towards downregulation of renal *AGT* expression between GDs 55 and 66 (*p* = 0.068). While changes in hepatic *REN* expression were not assessed due to low expression levels, expression of hepatic *ATP6AP2* (Figure 6c), a REN receptor, was significantly downregulated at GD 86 relative to all other timepoints (*p* < 0.001, *p* = 0.006, and *p* = 0.003 for GDs 55, 66, and 76, respectively), while renal *REN* expression (Figure 6d) was upregulated at GD 86 relative to GD 55 only (*p* = 0.002). An additional trend towards upregulation of renal *REN* expression was also observed between GDs 55 and 76 (*p* = 0.052).

Within each timepoint, treatment with MMI had a significant impact on hepatic *AGT* expression only, with expression in the MMI fetuses significantly decreased at both GDs 66 and 76 relative to the age‐matched CONs (*p* < 0.001 for both comparisons). A significant GD to treatment group interaction was observed, however, for both hepatic *AGT* and hepatic *ATP6AP2* expression (*p* = 0.001 and *p* = 0.008, respectively), while the interaction effect was nonsignificant for both renal *AGT* and renal *REN* expression.

### 3.6 Expression of ANGI‐converting enzymes

**FIGURE 7:**
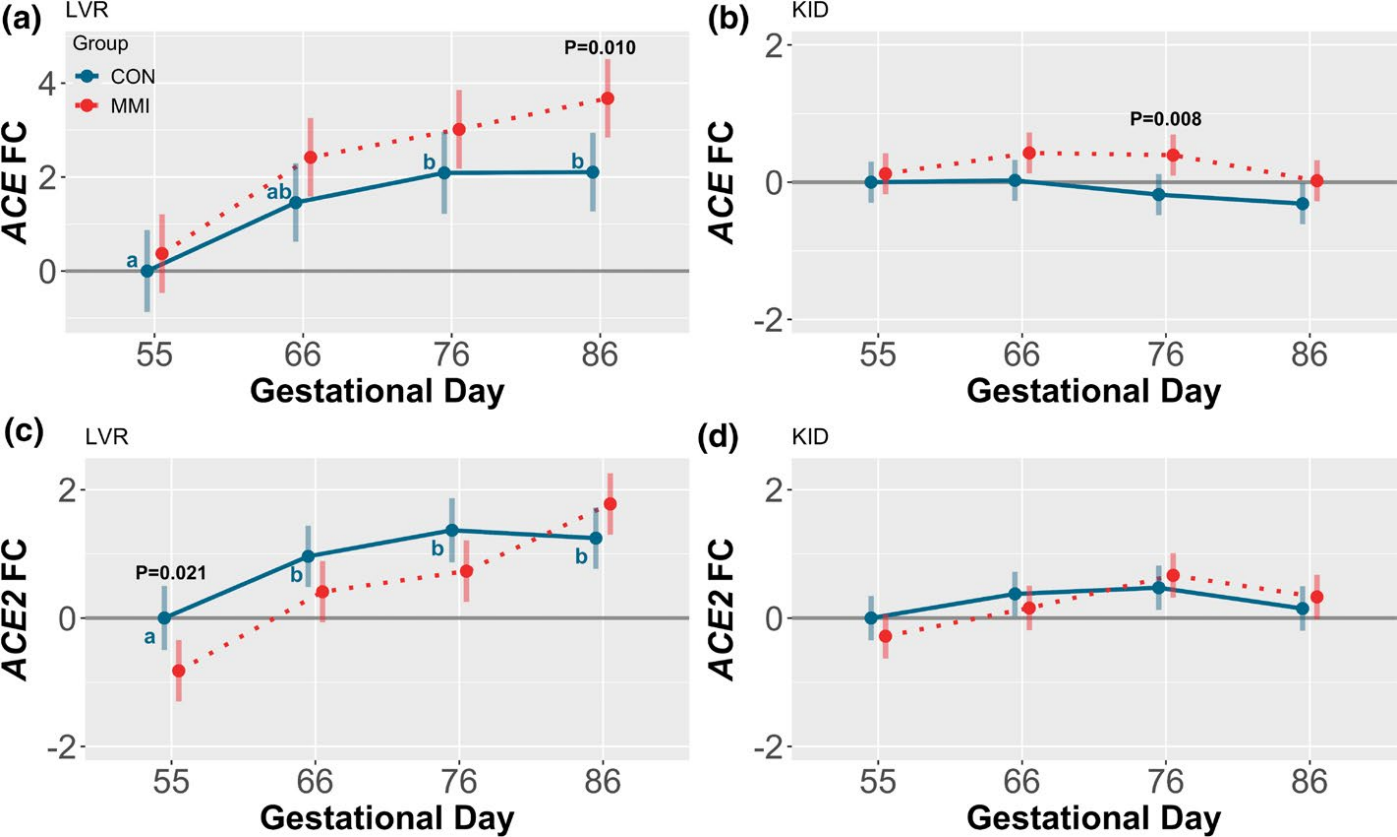
Gene expression of angiotensin I converting enzymes in fetal liver (LVR) and kidney (KID). Expression of (a, b) *ACE* and (c, d) *ACE2* in fetal (a, c) LVR and (b, d) KID tissue derived from fetuses (*n* = 11–12/group) at various gestational timepoints following maternal CON or MMI treatment for 21 days. All fold changes (FC) were calculated relative to the CON group at gestational day 55, with unique letter superscripts denoting statistically significant (*p* < 0.05) changes in gene expression in the CON group over time, and statistically significant differences between the CON and MMI group within each timepoint displayed numerically. Data were fit to a linear model including gestational day, treatment group, and the interaction, and are presented as estimated marginal means with 95% confidence intervals.

Ontogenic regulation of the two major ANGI‐converting enzymes, *ACE* and *ACE2*, was observed in the fetal LVR only (Figure 7a,c), with no impact of increasing gestational age on the expression of either gene in the KID (Figure 7b,d). Overall, the expression of both hepatic *ACE* and *ACE2* was upregulated as gestation progressed, with the expression of *ACE* significantly increased at GDs 76 and 86 relative to GD 55 (*p* = 0.007 and *p* = 0.005, respectively), and the expression of *ACE2* similarly upregulated at GDs 66, 76, and 86 relative to GD 55 (*p* = 0.042, *p* = 0.001, and *p* = 0.004, respectively).

MMI treatment resulted in tissue‐dependent temporal alterations in the expression of both of these genes, with the expression of hepatic *ACE* significantly increased in MMI fetuses at GD 86 relative to the age‐matched CON (*p* = 0.010), and renal expression of *ACE* significantly increased in MMI fetuses at GD 76 relative to CON (*p* = 0.008). Additionally, a trend was observed towards upregulation of *ACE* in the KID of MMI fetuses at GD 66 (*p* = 0.063). In contrast, MMI treatment resulted in a significant downregulation of hepatic *ACE2* expression at GD 55 only (*p* = 0.021), with an additional trend towards hepatic downregulation in the MMI group at GD 76 (*p* = 0.071). MMI treatment had no impact on renal *ACE2* expression at any of the studied timepoints. A significant GD by treatment group interaction was observed for hepatic expression of *ACE2* (*p* = 0.027), while the interaction effect was nonsignificant for hepatic *ACE*, renal *ACE*, and renal *ACE2* expression.

### 3.7 Expression of ANGII receptors

**FIGURE 8:**
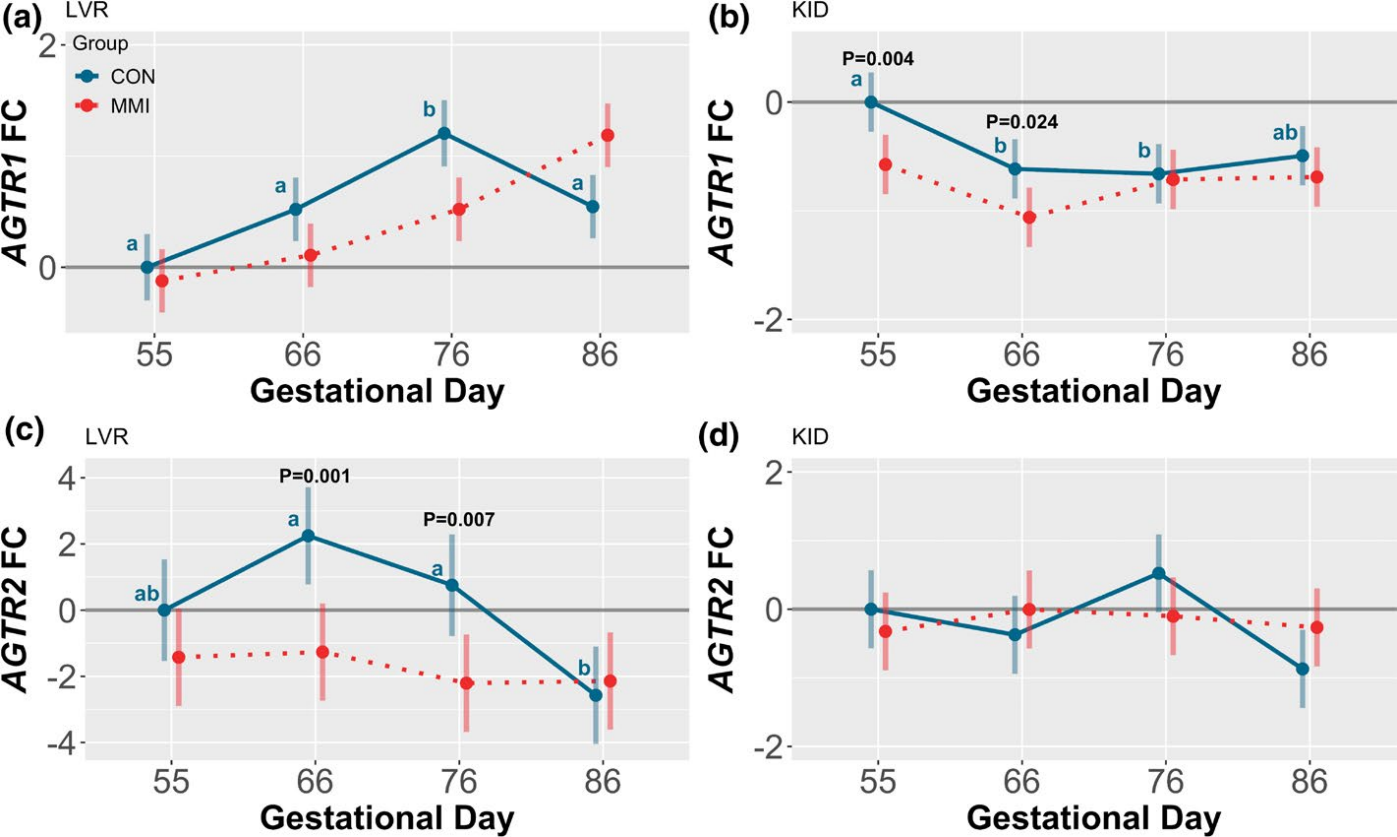
Gene expression of angiotensin II receptors in fetal liver (LVR) and kidney (KID). Expression of (a, b) *AGTR1* and (c, d) *AGTR2* in fetal (a, c) LVR and (b, d) KID tissue derived from fetuses (*n* = 11–12/group) at various gestational timepoints following maternal CON or MMI treatment for a period of 21 days. All fold changes (FC) were calculated relative to the CON group at gestational day 55, with unique letter superscripts denoting statistically significant (*p* < 0.05) changes in gene expression in the CON group over time. Statistically significant differences between the CON and MMI groups within each timepoint are displayed as *p*‐values. Data are presented as the estimated marginal means with 95% confidence intervals, as extracted from a linear model including gestational day, treatment group, and the interaction.

With regard to the CON groups, expression of the canonical ANGII receptor, *AGTR1*, was significantly impacted by gestational age in both the LVR and KID (Figure 8a,b), with hepatic expression of *AGTR1* significantly upregulated at GD 76 relative to all other timepoints (*p* < 0.001, *p* = 0.009, and *p* = 0.013 for GDs 55, 66, and 86, respectively), and renal expression of *AGTR1* significantly downregulated at GDs 66 and 76 relative to GD 55 only (*p* = 0.013 and *p* = 0.006, respectively). Additional trends towards upregulation of *AGTR1* were observed in the LVR at GDs 66 and 86 relative to GD 55 (*p* = 0.082 and *p* = 0.060, respectively), with a trend towards downregulation of *AGTR1* also seen in the KID at GD 86 relative to GD 55 (*p* = 0.075). In contrast to *AGTR1*, expression of *AGTR2* was ontogenically regulated in the LVR only (Figure 8c,d), with hepatic expression significantly decreased at GD 86 relative to GDs 66 and 76 (*p* < 0.001 and *p* = 0.015, respectively).

Within each timepoint, MMI had temporal impacts on renal expression of *AGTR1* and hepatic expression of *AGTR2*, with renal *AGTR1* expression decreased in the MMI groups relative to age‐matched CONs at GDs 55 and 66 (*p* = 0.004 and *p* = 0.024, respectively), and hepatic *AGTR2* expression decreased in the MMI groups relative to CONs at GDs 66 and 76 (*p* = 0.001 and *p* = 0.007, respectively). A significant GD by treatment group interaction was observed for both hepatic *AGTR1* and hepatic *AGTR2* expression (*p* < 0.001 and *p* = 0.043, respectively), with the interaction term nonsignificant for expression of both genes in the KID.

## 4 DISCUSSION

Fetal development is marked by significant ontogenic changes in critical endocrine systems, including the HPT axis and the RAS. While these systems are known to change systemically throughout gestation, with production of thyroid hormones increasing as gestation progresses (Brzezińska‐Slebodzińska & Slebodziński, 2004), and varying changes occurring in the RAS (Carbone et al., 1993; Olson et al., 1990), the tissue‐specific ontogenic regulation of these systems, as well as the interaction between them, remains unexplored in the porcine fetus. To this end, the objective of the present study was to first examine the ontogeny of the fetal ROID and of thyroid system and RAS‐related genes in the porcine fetal LVR and KID, and to subsequently assess the temporal impact of induced hypothyroidism on these measures. Using a temporal porcine model, we showed that the fetal thyroid system and local hepatic and renal RAS experience ontogenic changes throughout gestation, with these systems both significantly impacted by fetal hypothyroidism. Our findings suggest a complex relationship between the fetal thyroid system and fetal RAS that changes with both gestational age and studied tissue.

Prior studies have examined the morphological ontogeny of the porcine fetal ROID utilizing traditional staining methods and manual assessment under brightfield microscopy (Igbokwe & Ezeasor, 2015; van Fentener Vlissingen et al., 1983). Herein, we describe a fluorescent‐based lectin staining protocol that is conducive to automated morphological assessment, effectively allowing for quick, objective measurement of a large number of ROID follicles. One prior report in the pig indicates broad variability in the size of ROID follicles both pre‐ and postnatally (Igbokwe & Ezeasor, 2015). This heterogeneity has been widely accepted in a number of species (Sellitti & Suzuki, 2014) and warrants the need to measure many follicles in order to accurately assess morphology. In the present study, the average number of follicles quantified for each of the 24 CON fetuses was over 550, with these follicles detected over multiple images to prevent spatial bias. Our results regarding increasing follicular percent area and follicle area throughout gestation are consistent with prior reports in swine of a similar gestational age (van Fentener Vlissingen et al., 1983), while some of our reported parameters are novel, as assessment of more complex measures such as circularity and staining intensity would be difficult to quantitatively assess in standard brightfield microscopy.

Consistent with our prior qualitative work in the pig (Ison et al., 2023; Smith & Pasternak, 2025), the present study found that maternal administration of MMI leads to morphological changes in the fetal ROID, which is a classical indicator of hypothyroidism. Lectins bind to specific glycan structures (Kobayashi et al., 2014), making them useful for assessing the glycosylation status of various proteins. One previous study utilized Con A to assess protein glycosylation in ROID tissue of rats with altered thyroid hormone metabolism (Venugopalan et al., 2021), reporting decreased staining intensity in non‐euthyroid individuals. While the prior result was speculated to be attributed to altered glycosylation of thyroglobulin, which is a key protein required for thyroid hormone synthesis (Citterio et al., 2019), one limitation of lectin staining is that the exact protein identity and number of target proteins being bound cannot be easily elucidated, considering the diversity of protein glycosylation. Additionally, the species‐specific nature of glycosylation further complicates not only the identification of target proteins but the cross‐species use of specific lectins. In the present study, the decrease of parameters such as follicular percent area and detected follicle count in MMI fetuses is a likely indicator of abnormalities in either follicular protein abundance or glycosylation maturity. Both of these factors may jeopardize thyroid hormone production, which would be consistent with the decreased levels of thyroid hormones previously reported for the MMI fetuses utilized in the present study (Smith & Pasternak, 2025). Additionally, the presently observed decreases in follicular percent area and follicle circularity are consistent with what is observed in goitrous ROID histology in a variety of species, in which there is a breakdown of the normal spherical follicular structure and a loss of colloid (Fazioli et al., 2024; Tsujio et al., 2007). Collectively, these results support the utility of RCA120 staining for quantitatively assessing the morphological impact and severity of ROID dysfunction, as well as for examining ROID ontogeny throughout gestation.

Among CON animals, ontogenic changes in thyroid hormone binding protein and receptor expression throughout gestation may aid in regulating thyroid hormone bioavailability. The majority of circulating thyroid hormones exist bound to three hepatic‐derived binding proteins, being albumin, thyroid‐binding globulin, and transthyretin. Of the three, the latter two exhibit a higher affinity for thyroid hormones (Bartalena & Robbins, 1993) and are products of the *SERPINA7* and *TTR* genes, respectively. By binding to thyroid hormones and preventing their interaction with receptors, these two proteins aid in maintaining a consistent cellular bioavailability of thyroid hormones, despite potential physiological fluctuations (Bagga et al., 2023). In the fetal pig, increasing gestation is marked by ontogenic increases in thyroid hormone production (Brzezińska‐Slebodzińska & Slebodziński, 2004), which parallel the ontogenic increases in hepatic expression of *SERPINA7* and *TTR* observed in the present study. One could hypothesize that this increase in binding protein expression may be needed to prevent overactivity of thyroid hormones as production is significantly increased throughout gestation. In contrast, decreases in hepatic expression of *SERPINA7* in the MMI fetuses at GDs 66 and 76 may suggest a compensatory decrease in the percentage of bound thyroid hormones in an attempt to counteract the low circulating levels caused by MMI treatment. Alternatively, the observed decreases in *SERPINA7* expression may instead be explained by a failure of *SERPINA7* to follow a normal ontogenic trajectory in MMI fetuses, which appears rectified by GD 86. In addition to the present changes in gene expression, it has previously been reported that the expression of other genes regulating peripheral thyroid hormone metabolism, including those encoding key deiodinase enzymes and solute carriers, is dysregulated in the fetal LVR and KID in response to hypothyroidism (Ison et al., 2023). While not assessed in the present study, these changes may additionally play a role in the tissue‐specific response to hypothyroidism.

The current study presents both gene and protein‐level evidence of potential local RAS systems in the porcine fetal LVR and KID. While *REN* expression was too low to accurately assess in the LVR, the LVR not only expressed *AGT*, *ACE*, and *ACE2*, but was also found to have protein localization of these components. The presence or absence of hepatic *REN* expression has previously been shown to be species‐dependent (Fukamizu et al., 1994; Kon et al., 1998), but to the author's best knowledge, has yet to be directly assessed in the porcine fetal LVR. The absence of marked hepatic *REN* expression in the present study would suggest that if the porcine fetal LVR is to locally synthesize ANGII, it must do so by either utilizing non‐canonical enzymes to cleave AGT into ANGII, or by sequestering REN or prorenin from circulation to enhance local activity. Prior reports indicate chymase activity in the human LVR, which plays a role in local ANGII production (Hartl et al., 2023). Additionally, ATP6AP2, which was expressed in the LVR at all four time points utilized in this study, has previously been identified as a REN/prorenin receptor that increases local enzymatic activity (Jan Danser et al., 2007; Nguyen et al., 2002), and is known to be present in human LVR hepatocytes (Morimoto et al., 2021). Further, our Western Blot results provide evidence supporting intrahepatic cleavage of AGT, as the lower molecular weight band detected for AGT in the fetal LVR is consistent with a REN cleavage product. While we found that AGT in the fetal LVR was localized, as expected, throughout the cytoplasm of hepatocytes, interestingly, staining for ACE2 was also largely cytoplasmic in nature. Classically, ACE2 exists in two forms, being either membrane‐bound or circulating (Batlle et al., 2022). In contrast, the cytoplasmic localization observed in the present study may represent a truncated form of ACE2, which has previously been observed in the porcine LVR (Zhang et al., 2021) and would be consistent with the lower molecular weight band we observed in Western Blot.

In contrast to the LVR, the juxtaglomerular cells of the KID are the known canonical producers of REN (Faraggiana et al., 1982). Additionally, the present study found that the porcine fetal KID expresses and has protein localization of AGT, ACE, and ACE2, which would allow for completely independent ANGII and ANG‐(1–7) production. This is consistent with prior reports in other species, including humans, suggesting that both the fetal and mature KID have the capacity to locally produce all components needed for intrarenal ANGII production (Graciano et al., 2004; Tang et al., 2020). Unlike in humans, where nephrogenesis is completed during fetal life, nephrogenesis continues in the piglet until around 3 weeks postnatally (Zoetis & Hurtt, 2003). Treatment of piglets with ACE inhibitors during this early postnatal period has previously been shown to have negative developmental impacts on renal structure and function (Guron et al., 1998), although it is unclear whether this is a direct result of disruptions in the renal RAS, the systemic RAS, or a combination of the two. Considering our findings of local expression and production of RAS components in the porcine fetal KID, the pig may be a beneficial biomedical model for studying the impact of ACE inhibitors on not only the systemic RAS but also the local renal RAS, which is of particular interest considering the known teratogenic impact of ACE inhibitors on the human KID (Tabacova et al., 2003).

Among CON fetuses, ontogenic changes in the expression of many RAS genes were observed throughout gestation. The most marked ontogenic alteration occurred in the KID, with the expression of renal *AGT* steadily decreasing as gestation progressed, leading to an almost 4‐fold expression decrease at GD 86 relative to GD 55. This decrease in expression over time may suggest that local renal AGT production is more important during early gestation, after which the KID, and potentially other tissues, becomes increasingly dependent on hepatic‐derived AGT. This is consistent with prior reports suggesting that in the fetal rat, the LVR is not the primary source of AGT, but rather, AGT is synthesized locally in a variety of fetal tissues including the KID (Gomez et al., 1988). However, the existence of renal *AGT* expression in the present study contrasts with reports in fetal sheep in which *AGT* mRNA was undetectable in renal cortical tissue throughout gestation (Olson et al., 1990), effectively suggesting that tissue production and regulation of AGT may be species‐specific. In addition to changes in renal *AGT* expression, the present study observed that renal *REN* expression increased with advancing gestational age, which is consistent with prior work in the fetal sheep (Carbone et al., 1993) and may suggest an overall increase in RAS activity throughout gestation considering that REN catalyzes the rate‐limiting step of ANGII production. Lastly, ontogenic increases in hepatic, but not renal, *ACE* and *ACE2* expression were observed between GDs 55 and 86, albeit with minor overall fold changes. Consistent with the stability of *ACE* expression we observed in the KID, one of the only previous papers discussing RAS ontogeny in the fetal pig reported that levels of pulmonary and renal ACE do not exhibit marked changes throughout late gestation, despite being altered in adulthood relative to fetal levels (Forhead et al., 2001). The changes observed postnatally in the aforementioned study were observed to be tissue‐specific, consistent with our findings regarding the tissue‐dependent regulation of both ACE enzymes in mid‐ to late gestation.

Fetal hypothyroidism was found to have temporal and tissue‐specific effects on the expression of many RAS genes including *AGT*, *ACE*, *ACE2*, *AGTR1*, and *AGTR2*. In mature humans, hypothyroidism is known to increase the risk for developing hypertension (Danzi & Klein, 2003). Additionally, congenitally hypothyroid rats experience hypertension in adulthood (Santos et al., 2012), suggesting that in utero deficits in thyroid hormone production may lead to postnatal programming of hemodynamics. If regulated by the RAS, increases in blood pressure would require either a functional increase in the canonical ANGII‐forming pathway or a decrease in the production and activity of ANG‐(1–7). In the present study, there is some evidence of an enzymatic upregulation in canonical RAS activity in hypothyroid fetuses, as indicated by temporal increases in hepatic and renal *ACE* expression, with a temporal decrease in hepatic *ACE2* expression at GD 55 potentially indicating a suppression of the non‐canonical ANG‐(1–7) pathway. However, a number of prior studies examining the consequences of fetal hypothyroidism on the RAS have suggested a suppression, rather than activation, of the canonical RAS. This is marked by decreased fetal renal *REN* expression (Chen et al., 2005), temporally decreased fetal pulmonary and renal ACE levels (Forhead & Fowden, 2002), and decreased postnatal ANGII concentrations following congenital hypothyroidism (Matilla et al., 1993). Additionally, hypothyroidism in fetal sheep is known to cause a reduction in blood pressure (Chen et al., 2005), indicating fetal hypotension, rather than hypertension. This suggests either a species‐specific response of hemodynamics to hypothyroidism or a varying response between mature and fetal life. In light of these apparent contradictions, more research is needed to determine the exact relationship between hypothyroidism, the RAS, and blood pressure regulation, which will ultimately require functional studies in relevant biomedical models such as the pig.

## 5 CONCLUSIONS

Collectively, our results provide insights into the ontogeny of the porcine fetal thyroid system and local hepatic and renal RAS and also elucidate the temporal nature of the interaction between these systems. The follicles in the fetal ROID were found to increase in area over time, whilst decreasing in circularity, with follicular morphology significantly impacted by fetal hypothyroidism at all studied timepoints. Expression of many thyroid and RAS‐related genes was found to be temporally regulated throughout gestation, and also significantly dysregulated by fetal hypothyroidism. The impact of fetal hypothyroidism on gene expression was found to be dependent on both gestational age and tissue, suggesting a complex relationship between the fetal thyroid system and local RAS. Future research should assess the biological significance of local RAS systems in the porcine fetal LVR and KID to determine their importance for normal development, as well as seek to understand how disruptions in these local RAS, as can result from fetal hypothyroidism, may be teratogenic. Additionally, an investigation of the postnatal consequences of congenital hypothyroidism on the RAS is warranted to determine the potential for in utero programming of this system, which may lead to long‐term health detriments.

## AUTHOR CONTRIBUTIONS

J.A.P. and A.A.S. conceived of the study and initial hypothesis. A.A.S. conducted the laboratory procedures, performed the statistical analyses, and drafted the manuscript, which was reviewed and approved by J.A.P.

## FUNDING INFORMATION

This study was supported by the intramural research program of the U.S. Department of Agriculture, National Institute of Food and Agriculture, Agriculture and Food Research Initiative [2023‐67015‐39338]. The findings and conclusions have not been formally disseminated by the U.S. Department of Agriculture and should not be construed to represent any agency determination or policy.

## CONFLICT OF INTEREST STATEMENT

The authors declare no conflict of interest.

## Supporting information


Figure S1.

## Data Availability

The data that support the findings of this study are available from the corresponding author upon reasonable request.
