# Supplementary material for: Gestational age and fetal hypothyroidism Alter the porcine thyroid and local hepatic and renal renin‐angiotensin systems
Source: Physiol Rep. 2025 Sep 12;13(17):e70565. doi: 10.14814/phy2.70565 (PMC12431595; doi:10.14814/phy2.70565)
Supplement: Supplementary file 1 — Figure S1. [file PHY2-13-e70565-s001.docx]

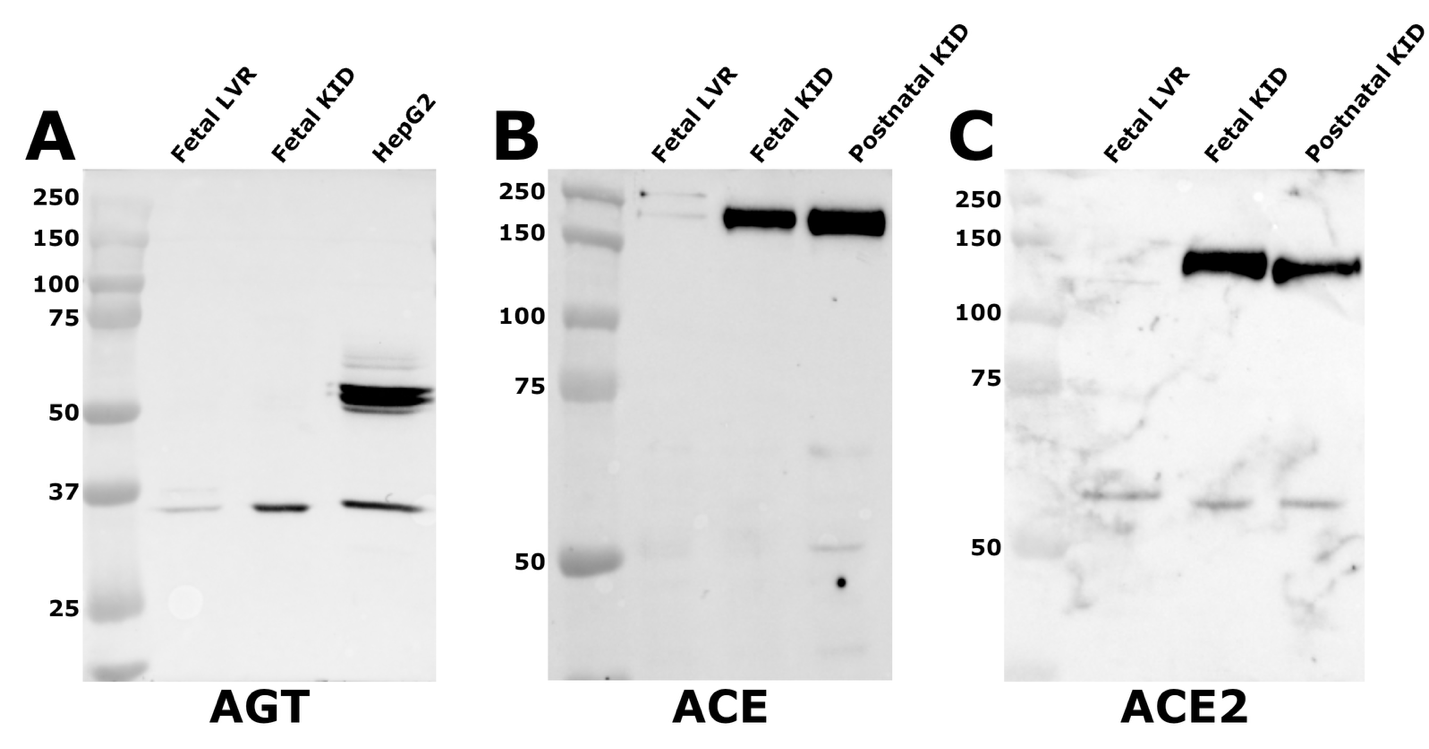


**Supplemental Figure S1:** *Western Blots showing target specificity of antibodies used for IHF.* (A) 40 µg porcine fetal liver (LVR) protein, 40 µg porcine fetal kidney (KID) protein, and 30 µg HepG2 protein was run down a standard 10% SDS-PAGE gel and incubated overnight at 4 °C in 1 µg/mL AGT antibody. (B) 48 µg porcine fetal LVR protein, 48 µg/mL porcine fetal KID protein, and 48 µg porcine postnatal KID protein was run down a standard 8% SDS-PAGE gel and incubated overnight at 4 °C in 1 µg/mL ACE antibody. (C) 48 µg porcine fetal LVR protein, 48 µg/mL porcine fetal KID protein, and 48 µg porcine postnatal KID protein was run down a standard 8% SDS-PAGE gel and incubated overnight at 4 °C in 0.2 µg/mL ACE2 antibody. All detection was performed using ECL substrate, with molecular weight markers (kDa) shown in the leftmost lane of each gel.


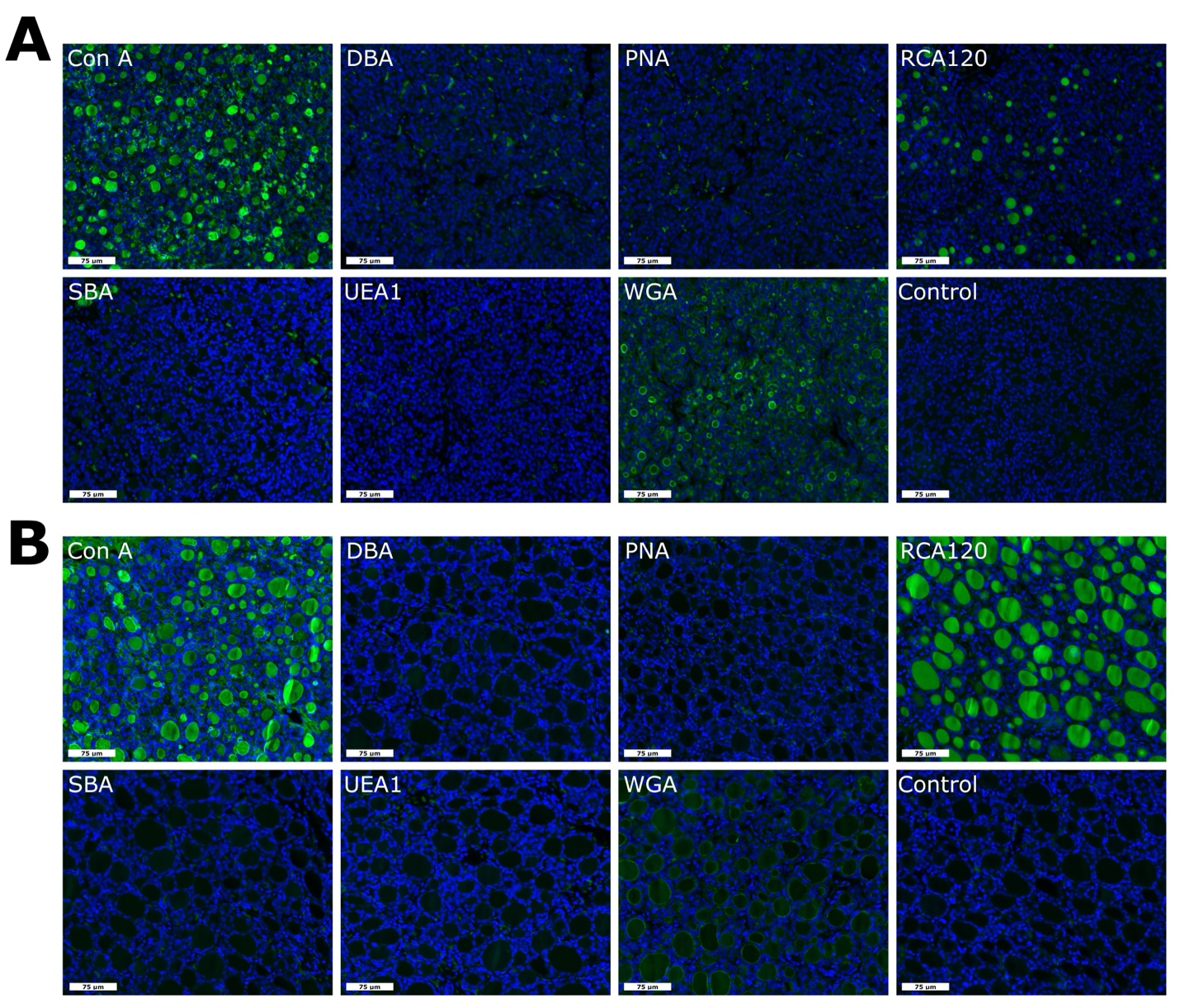


**Supplemental Figure S2:** *Preliminary test of lectin staining patterns in fetal thyroids.* Representative images showing staining patterns of different Fluorescein-conjugated lectins including Con A, DBA, PNA, RCA120, SBA, UEA1, and WGA in fetal thyroid tissue derived from CON fetuses at (A) gestational day 55 and (B) gestational day 86. Staining for each lectin is shown in green, with DAPI shown as a counterstain and colored blue. Lectin staining was confirmed positive or negative relative to a control sample that was incubated in PBS only, with Con A, RCA120, and WGA exhibiting marked positive staining at both gestational days.


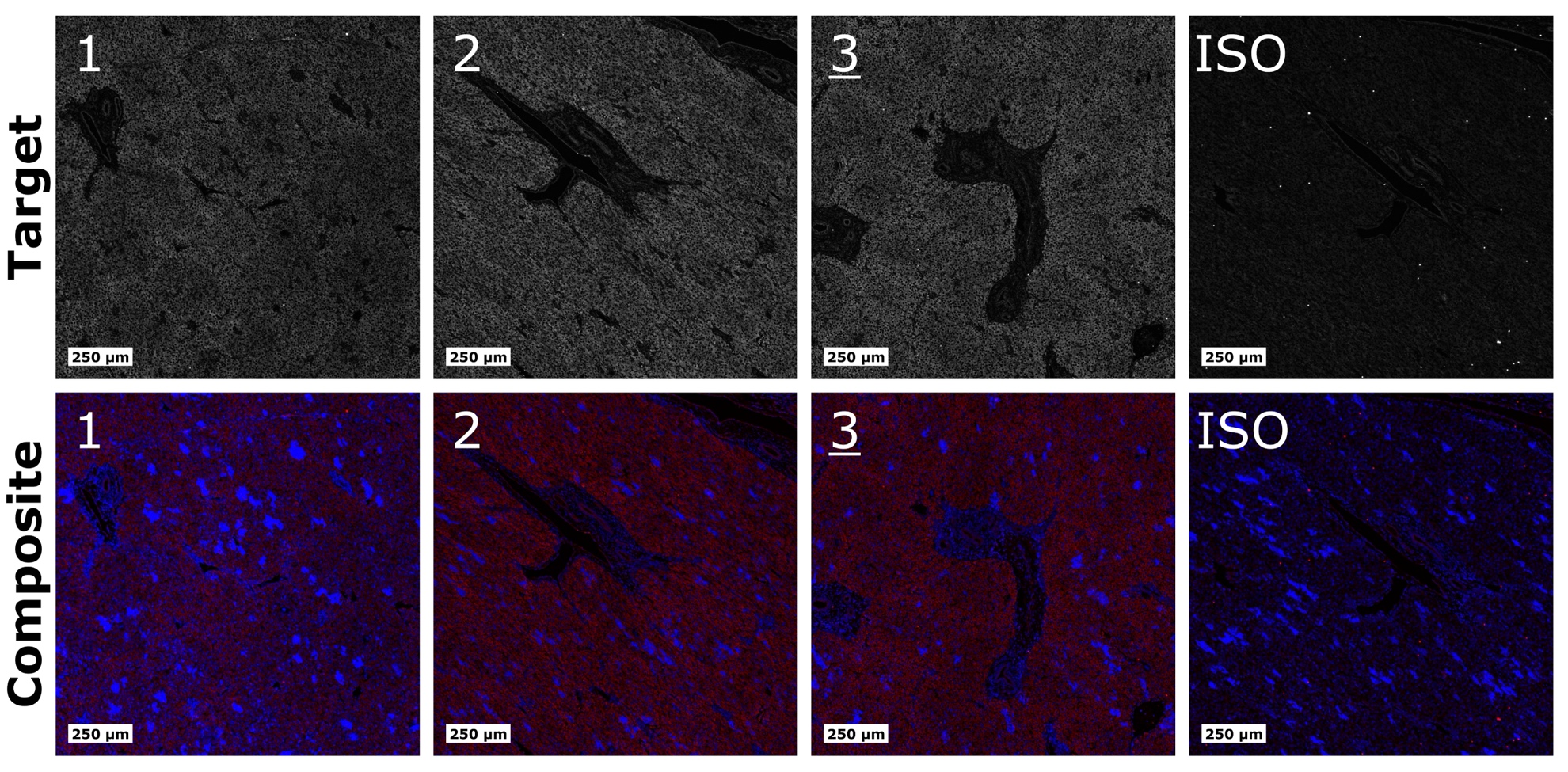


**Figure S3:** *Immunohistofluorescent staining of AGT in fetal liver.* IHF images showing cellular localization of AGT in fetal liver tissue derived from three porcine fetuses at day 96 of gestation. Monochromatic images numbered 1-3 represent AGT, with an isotype control (ISO) shown in the rightmost image. AGT or the equivalent ISO are represented in red in the composite images, with DAPI shown as a counterstain and colored blue. The underlined number represents the image that was selected as representative and included in the main figure.


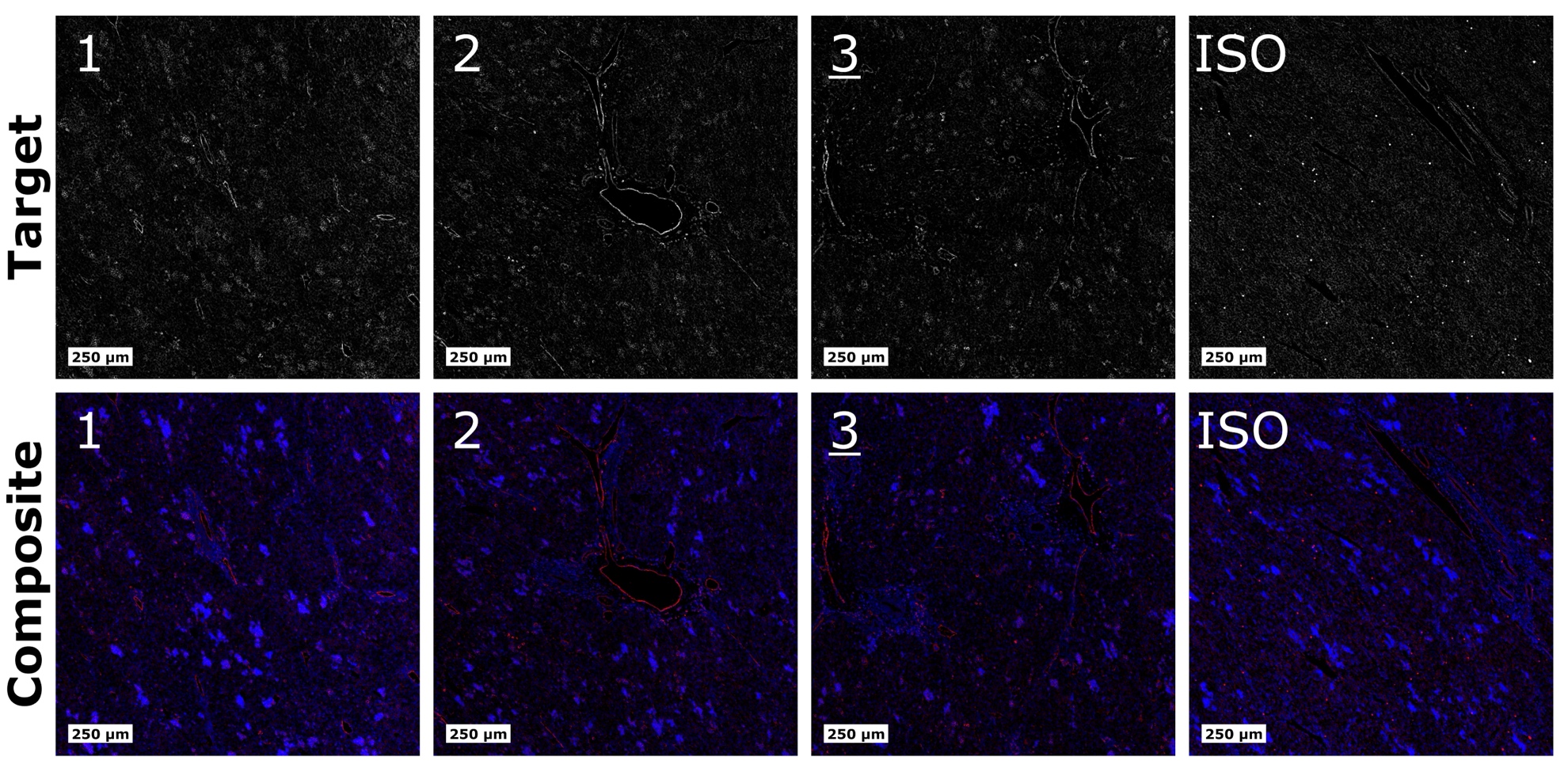


**Figure S4:** *Immunohistofluorescent staining of ACE in fetal liver.* IHF images showing cellular localization of ACE in fetal liver tissue derived from three porcine fetuses at day 96 of gestation. Monochromatic images numbered 1-3 represent ACE, with an isotype control (ISO) shown in the rightmost image. ACE or the equivalent ISO are represented in red in the composite images, with DAPI shown as a counterstain and colored blue. The underlined number represents the image that was selected as representative and included in the main figure.


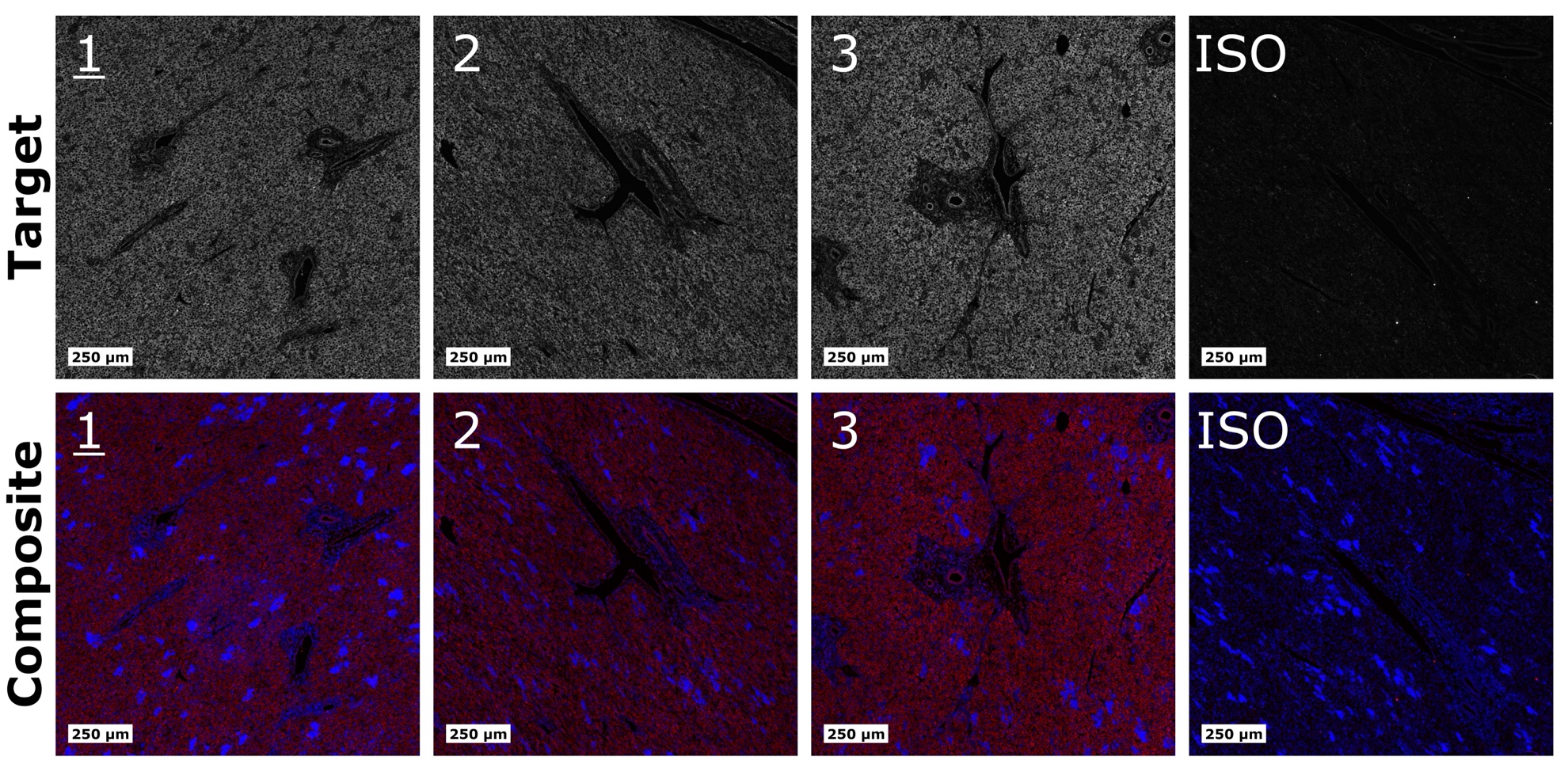


**Figure S5:** *Immunohistofluorescent staining of ACE2 in fetal liver.* IHF images showing cellular localization of ACE2 in fetal liver tissue derived from three porcine fetuses at day 96 of gestation. Monochromatic images numbered 1-3 represent ACE2, with an isotype control (ISO) shown in the rightmost image. ACE2 or the equivalent ISO are represented in red in the composite images, with DAPI shown as a counterstain and colored blue. The underlined number represents the image that was selected as representative and included in the main figure.


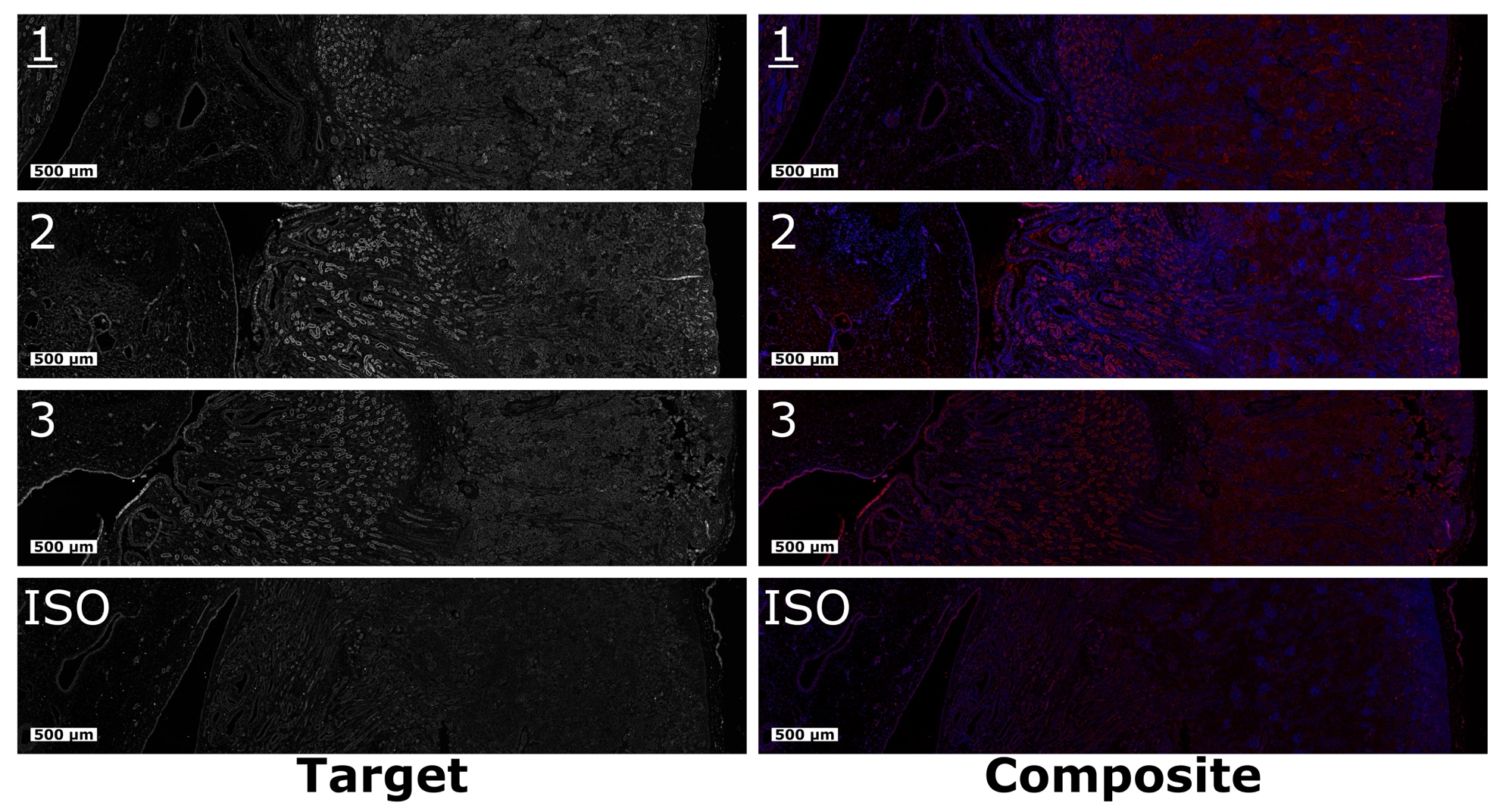


**Figure S6:** *Immunohistofluorescent staining of AGT in fetal kidney.* IHF images showing cellular localization of AGT in fetal kidney tissue derived from three porcine fetuses at day 96 of gestation. Monochromatic images numbered 1-3 represent AGT, with an isotype control (ISO) shown in the bottom image. AGT or the equivalent ISO are represented in red in the composite images, with DAPI shown as a counterstain and colored blue. The underlined number represents the image that was selected as representative and included in the main figure.


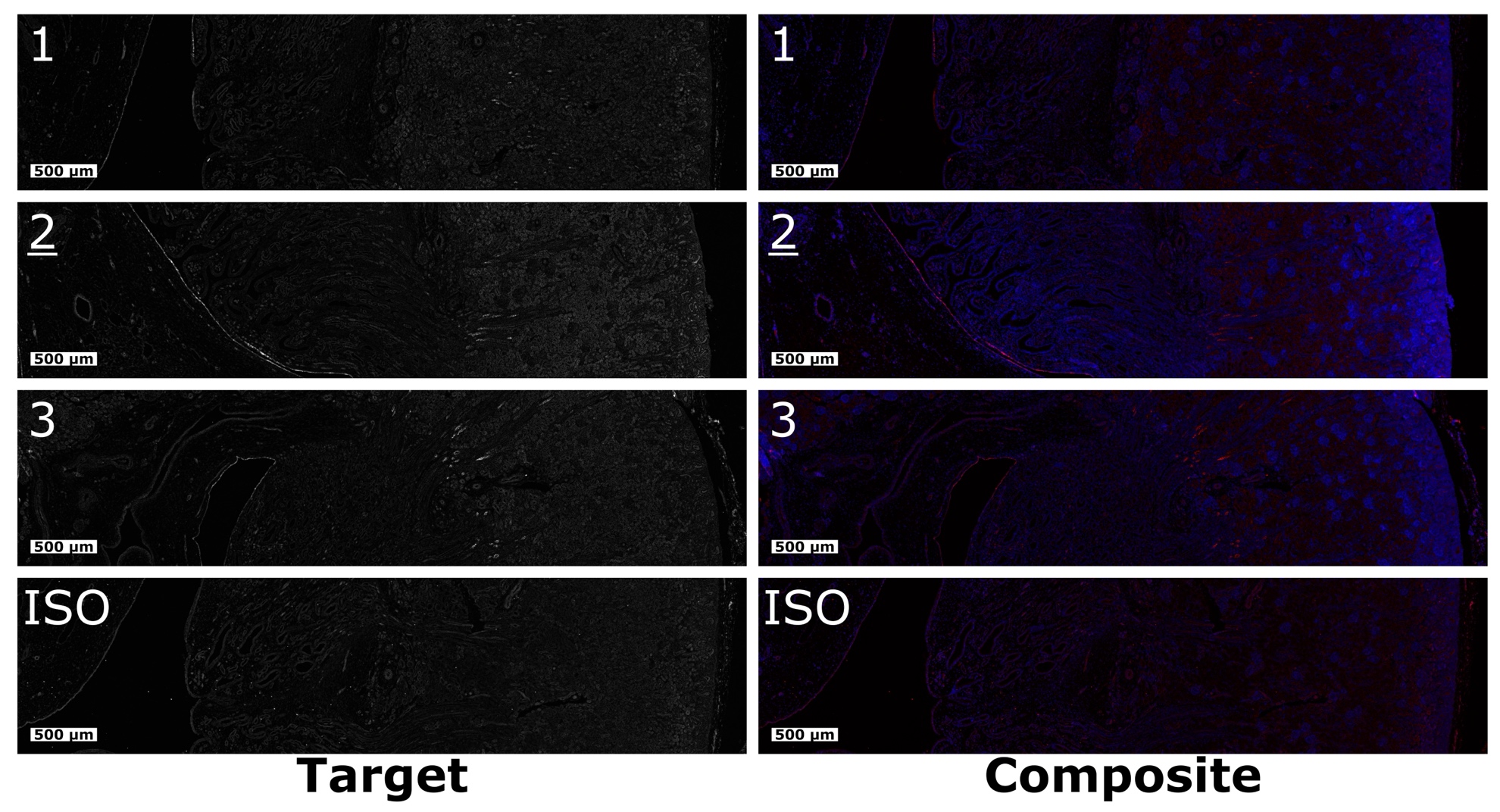


**Figure S7:** *Immunohistofluorescent staining of ACE in fetal kidney.* IHF images showing cellular localization of ACE in fetal kidney tissue derived from three porcine fetuses at day 96 of gestation. Monochromatic images numbered 1-3 represent ACE, with an isotype control (ISO) shown in the bottom image. ACE or the equivalent ISO are represented in red in the composite images, with DAPI shown as a counterstain and colored blue. The underlined number represents the image that was selected as representative and included in the main figure.


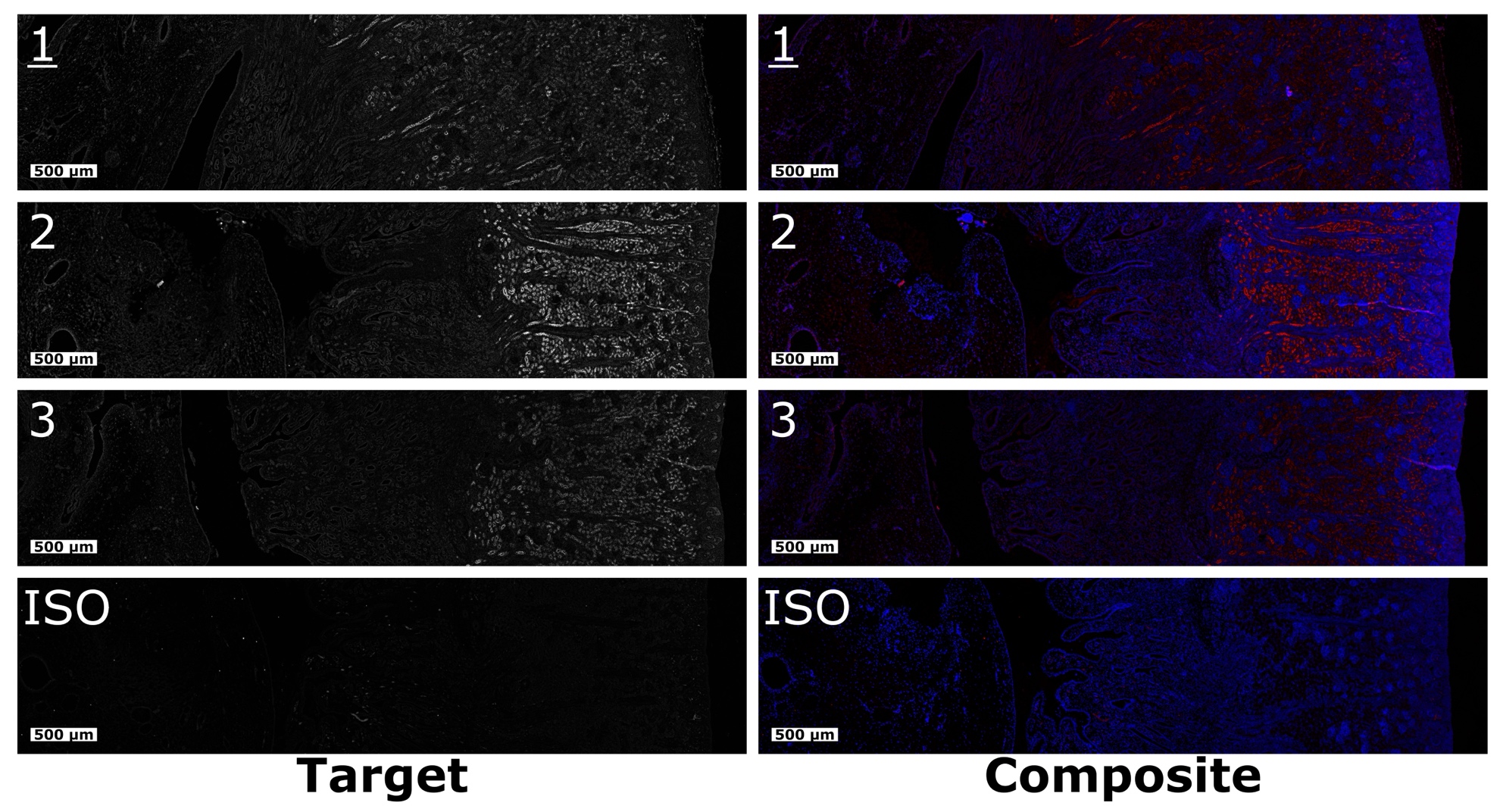


**Figure S8:** *Immunohistofluorescent staining of ACE2 in fetal kidney.* IHF images showing cellular localization of ACE2 in fetal kidney tissue derived from three porcine fetuses at day 96 of gestation. Monochromatic images numbered 1-3 represent ACE2, with an isotype control (ISO) shown in the bottom image. ACE2 or the equivalent ISO are represented in red in the composite images, with DAPI shown as a counterstain and colored blue. The underlined number represents the image that was selected as representative and included in the main figure.
